# Generation of a High Number of Healthy Erythroid Cells from Gene-Edited Pyruvate Kinase Deficiency Patient-Specific Induced Pluripotent Stem Cells

**DOI:** 10.1016/j.stemcr.2015.10.002

**Published:** 2015-11-05

**Authors:** Zita Garate, Oscar Quintana-Bustamante, Ana M. Crane, Emmanuel Olivier, Laurent Poirot, Roman Galetto, Penelope Kosinski, Collin Hill, Charles Kung, Xabi Agirre, Israel Orman, Laura Cerrato, Omaira Alberquilla, Fatima Rodriguez-Fornes, Noemi Fusaki, Felix Garcia-Sanchez, Tabita M. Maia, Maria L. Ribeiro, Julian Sevilla, Felipe Prosper, Shengfang Jin, Joanne Mountford, Guillermo Guenechea, Agnes Gouble, Juan A. Bueren, Brian R. Davis, Jose C. Segovia

**Affiliations:** 1 Hematopoietic Innovative Therapies Division, Centro de Investigaciones Energéticas, Medioambientales y Tecnológicas (CIEMAT), Centro de Investigaciones Biomédicas en Red de Enfermedades Raras (CIBERER), Madrid 28040, Spain; 2 Advanced Therapies Mixed Unit, Instituto de Investigación Sanitaria-Fundación Jiménez Díaz (IIS-FJD, UAM), Madrid 28040, Spain; 3 Center for Stem Cell and Regenerative Medicine, Brown Foundation Institute of Molecular Medicine, University of Texas Health Science Center, Houston, TX 77030, USA; 4 Institute of Cardiovascular and Medical Sciences, University of Glasgow, Glasgow G12 8QQ, UK; 5 Cellectis, Paris 75013, France; 6 Agios Pharmaceuticals, Cambridge, MA 02139-4169, USA; 7 Hematology and Cell Therapy, Clinica Universidad de Navarra and CIMA, Pamplona 31008, Spain; 8 JST PRESTO and Ophthalmology, Keio University, Tokyo 108-8345, Japan; 9 Histocompatibility and Molecular Biology Laboratory, Centro de Transfusion de Madrid, Madrid 28032, Spain; 10 Serviço de Hematologia, Centro Hospitalar e Universitario de Coimbra, Coimbra 3000-075, Portugal; 11 Hospital Universitario Niño Jesús, Madrid 28009, Spain

## Abstract

Pyruvate kinase deficiency (PKD) is a rare erythroid metabolic disease caused by mutations in the *PKLR* gene. Erythrocytes from PKD patients show an energetic imbalance causing chronic non-spherocytic hemolytic anemia, as pyruvate kinase defects impair ATP production in erythrocytes. We generated PKD induced pluripotent stem cells (PKDiPSCs) from peripheral blood mononuclear cells (PB-MNCs) of PKD patients by non-integrative Sendai viral vectors. PKDiPSCs were gene edited to integrate a partial codon-optimized R-type pyruvate kinase cDNA in the second intron of the *PKLR* gene by TALEN-mediated homologous recombination (HR). Notably, we found allele specificity of HR led by the presence of a single-nucleotide polymorphism. High numbers of erythroid cells derived from gene-edited PKDiPSCs showed correction of the energetic imbalance, providing an approach to correct metabolic erythroid diseases and demonstrating the practicality of this approach to generate the large cell numbers required for comprehensive biochemical and metabolic erythroid analyses.

## Highlights

• Patient-specific PKDiPSCs are generated from PB-MNCs by a non-integrative system
• PKDiPSCs are gene edited to insert a partial co-RPK in the *PKLR* locus mediated by TALEN
• An SNP in the homology arm leads to allele-specific homologous recombination
• Gene-edited PKDiPSCs generate a high number of metabolically corrected erythroid cells

## Teaser

Patient-specific induced pluripotent stem cells (iPSCs) are the perfect platform to study erythroid metabolic diseases and test innovative treatments. Segovia, Quintana-Bustamante, and colleagues showed the correction of pyruvate kinase deficiency (PKD) by combining iPSC and gene-editing technologies and provide an approach to generate the large number of erythroid cells required for comprehensive biochemical and metabolic analyses of this disease and its treatment.

## Introduction

Pyruvate kinase deficiency (PKD; OMIM: 266200) is a rare metabolic erythroid disease caused by mutations in the *PKLR* gene, which codes the R-type pyruvate kinase (RPK) in erythrocytes and L-type pyruvate kinase (LPK) in hepatocytes. Pyruvate kinase (PK) catalyzes the last step of glycolysis, the main source of ATP in mature erythrocytes (Zanella et al., 2007). PKD is an autosomal-recessive disease and the most common cause of chronic non-spherocytic hemolytic anemia. The disease becomes clinically relevant when RPK activity decreases below 25% of the normal activity in erythrocytes. PKD treatment is based on supportive measures, such as periodic blood transfusions and splenectomy. The only definitive cure for PKD is allogeneic bone marrow transplantation (Suvatte et al., 1998, Tanphaichitr et al., 2000). However, the low availability of compatible donors and the risks associated with allogeneic bone marrow transplantation limit its clinical application. Transplantation of gene-corrected autologous hematopoietic progenitors might solve these problems. We have developed different gamma-retroviral and lentiviral vectors to correct a mouse PKD model (Meza et al., 2009), and their efficacy is currently being tested in hematopoietic progenitors from PKD patients (M. Garcia-Gomez et al., personal communication). However, the main drawback of current gene therapy approaches based on retro-/lentiviral vectors is the random integration of transgenes, which can promote insertional mutagenesis by disrupting tumor suppressor genes or *cis*-activating proto-oncogenes (Cavazza et al., 2013).

Over the last few years, gene editing by homologous recombination (HR) has been widely used in human cells to avoid undesirable transgene insertion. HR efficacy is very limited in human cells, estimated at one HR event per 10^6^ cells; however, the potential application of HR in human cells has been enhanced considerably by sequence-specific DNA nucleases (Carroll, 2011, Porteus and Carroll, 2005). Three different gene-editing strategies can be applied: gene correction, where a mutation is exchanged directly by the wild-type sequence; knockin, where a partial cDNA is inserted in the target locus to express a chimeric mRNA formed by endogenous first exons and partial cDNA under the endogenous promoter control; and safe harbor, in which the transgene is inserted by HR in a safe place in the genome, such as AAVS1 or CCR5 loci (Garate et al., 2013).

Concurrent with the application of gene editing in human cells, the generation of human induced pluripotent stem cells (iPSCs) was described (Takahashi et al., 2007, Yu et al., 2007). iPSCs possess properties of self-renewal and pluripotency that are similar to those of embryonic stem cells (ESCs), but potential alloreactivity and ethical issues associated with human ESCs are avoided. The wide reproducibility of the iPSC technology, independent of cell type and reprogramming methods, has established their great potential for future cell therapies. Additionally, patient- or disease-specific iPSCs are becoming established as in vitro systems to model diseases and to explore new therapeutic approaches. Reprogramming of easily accessible cell sources such as skin fibroblasts (Park et al., 2008), keratinocytes (Aasen et al., 2008), or even peripheral blood mononuclear cells (PB-MNCs) (Loh et al., 2009, Ye et al., 2009) has been described, and many efforts are being made to improve the safety and efficacy of the reprogramming method. Recently, iPSC generation by a Sendai viral vector platform (SeV) (Fusaki et al., 2009, Nishimura et al., 2011), even from blood cells (Nishishita et al., 2011, Seki et al., 2010), has been described as a non-integrative and highly efficient platform.

The correction of patient-specific iPSCs by homologous recombination has been explored in different pathologies (Garate et al., 2013, Karakikes et al., 2015, Rio et al., 2014, Sebastiano et al., 2011, Song et al., 2015), demonstrating its feasibility and setting up gene editing for other stem cells. Herein, we have assessed the combination of cell reprogramming and gene editing for PKD correction as a first example of the possible application of these advanced technologies to metabolic diseases affecting the erythroid lineage. PKD patient-specific iPSCs were efficiently generated from PB-MNCs by an SeV non-integrative system. The *PKLR* gene was edited by PKLR transcription activator-like effector nucleases (TALENs) to introduce a partial codon-optimized cDNA in the second intron by HR. Surprisingly, we found allelic specificity in the HR induced by the presence of a single nucleotide exchange (SNP), demonstrating the potential to select the allele to be corrected. Significantly, a high number of erythroid cells derived from PKDiPSCs was generated and displayed the energetic imbalance characteristic of PKD patients, which was corrected after gene editing.

## Results

### Generation of Integration-free Specific iPSCs Derived from the Peripheral Blood of PKD Patients

First, to evaluate the potential use of PB-MNCs as a cell source to be reprogrammed to iPSCs by the non-integrative SeV, we analyzed the susceptibility of these cells to SeV. PB-MNCs were expanded in the presence of specific cytokines (stem cell factor [SCF], thrombopoietin [TPO], FLT3L, granulocyte colony-stimulating factor [G-CSF], and IL-3) to promote the maintenance and proliferation of hematopoietic progenitors and myeloid-committed cells for 4 days. Cells were then infected with an SeV encoding for the Azami green fluorescent marker. Five days later, the transduction of hematopoietic progenitor (CD34^+^), myeloid (CD14^+^/CD15^+^), and lymphoid T (CD3^+^) and B (CD19^+^) cells was evaluated by flow cytometry. Although the majority of cells in the culture expressed T or B lymphoid markers, a reduced proportion of them (10% of T cells, 3% of B cells) expressed Azami green. In contrast, 54% of the myeloid cells and 76% of the hematopoietic progenitors present in the culture were positive for the fluorescent marker (data not shown), demonstrating that SeV preferentially transduces the less abundant hematopoietic progenitors and myeloid cells under these culture conditions.

**Figure 1:**
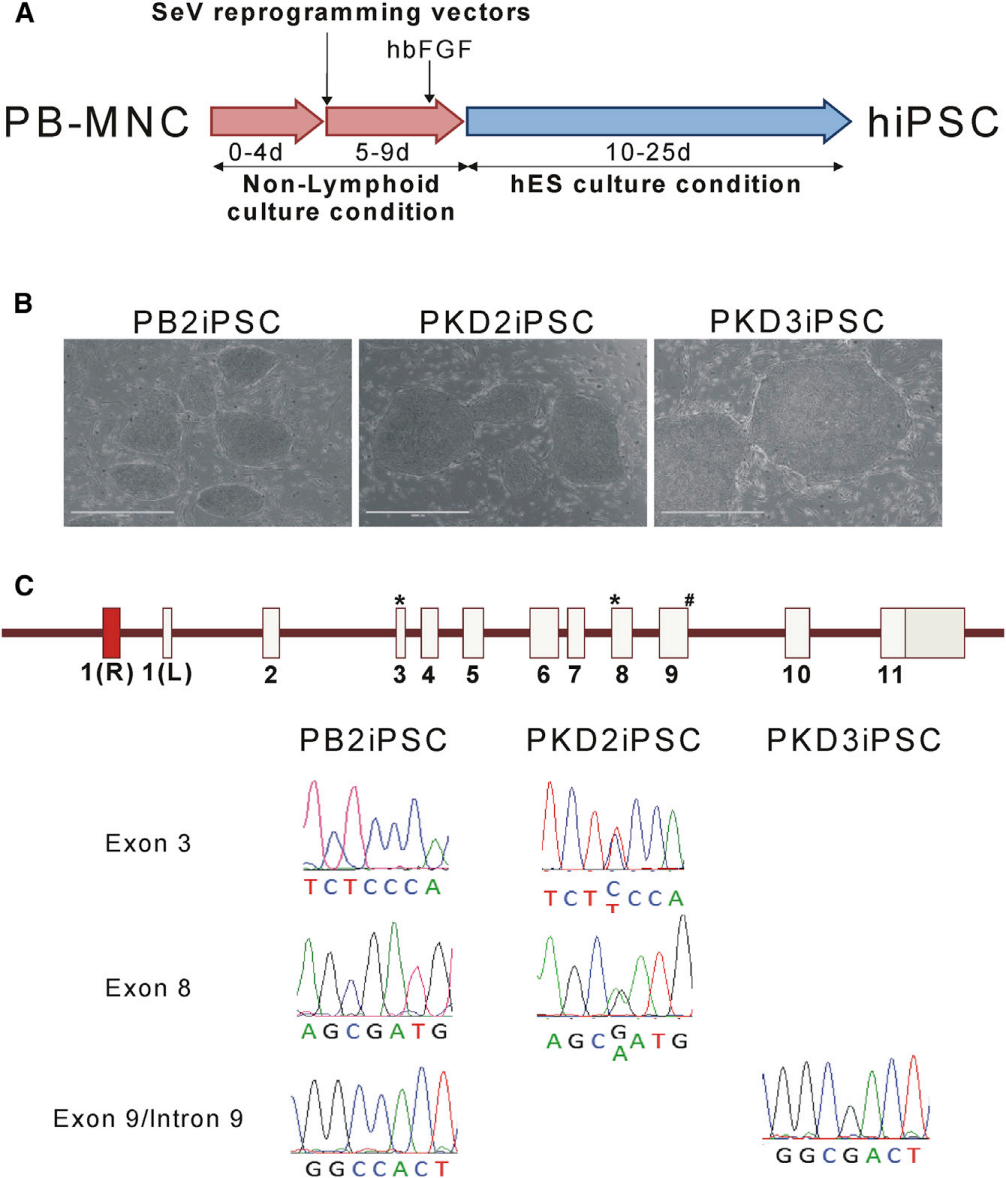
PB-MNC Reprogramming by SeV PB-MNCs from healthy donors and PKD patients were reprogrammed by SeV expressing OCT4, SOX2, KLF4, and cMYC mRNAs. Several lines from a healthy donor (PB2iPSC), patient PKD2 (PKD2iPSC), and patient PKD3 (PKD3iPSC) were isolated, expanded, and characterized. (A) Diagram of the reprogramming protocol. (B) Representative microphotographs of different iPSC lines derived from PB2 MNC, PKD2 MNC, or PKD3 MNC. Scale bars represent 200 μm. (C) Sanger sequencing of each patient-specific mutation in the *PKLR* gene in PB2iPSC, PKD2iPSC, and PKD3iPSC. ^∗^Mutations present in patient PKD2. ^#^Mutation present in patient PKD3. See also Figures S1 and S2.

This transduction protocol was then used to reprogram PB-MNCs from healthy donors and PKD patients by SeV encoding the four “Yamanaka” reprograming factors (OCT3/4, KLF4, SOX2, and c-MYC; Figure 1A). ESC-like colonies were obtained from one healthy donor (PB2) and from samples from two PKD patients (PKD2 and PKD3) PB-MNCs. Up to 20 ESC-like colonies derived from PB2, 100 from PKD2 and 50 from PKD3 were isolated and expanded (Figure 1B). The complete reprogramming of the different established lines toward embryonic stem (ES)-like cells was evaluated (Figures S1A–S1C). RT-PCR gene expression array verified a similar expression level of the main genes involved in pluripotency and self-renewal in our reprogramed cells and in the reference human ESC line H9 (Figures S1A–S1C). The ES markers *OCT3/4, SSEA4*, and *Tra-1-60* were also corroborated by fluorescence-activated cell sorting (FACS) and immunofluorescence (Figures S1A–S1C). Unmethylated status of *NANOG* and *SOX2* promoters was confirmed by pyrosequencing. *NANOG* promoter was strongly demethylated in lines derived from PB2, PKD2, and PKD3. Surprisingly, the *SOX2* promoter was already unmethylated in PB-MNCs (Figure S1D). Furthermore, the pluripotency of these lines derived from PB-MNCs was affirmed by their ability to generate teratomas into NOD.Cg-PrkdcscidIL2rgtm/Wjl/SzJ (NSG) mice, where all the mice injected developed teratomas showing tissues from the three different embryonic layers (Figures S1A–S1C). These data confirmed the reprogrammed lines as bona fide iPSC lines denoted as PB2iPSC, PKD2iPSC, and PKD3iPSC. Additionally, the presence of the wild-type (WT) sequence or patient specific mutations in the different human iPSC lines generated was confirmed by Sanger sequencing of the corresponding genome loci (Figure 1C). PKD2iPSC showed the two heterozygous mutations in exon 3 (359C > T) and exon 8 (1168G > A), and PKD3iPSC carried the homozygous mutation in the splicing donor sequence of exon 9/intron 9 (IVS9(+1)G > C) characterized in the patients. These mutations could not be detected in PB2iPSC, which showed the expected WT sequences (Figures 1C).

To confirm the absence of ectopic reprogramming gene expression, we analyzed the disappearance of SeV vectors in the generated iPSCs. The presence of the ectopic proteins could be tracked by the persistence of the fluorescent marker, as the SeV expressing Azami green was co-transduced together with the reprogramming vectors. Azami green expression was only detected in non-reprogramed, fibroblast-like cells in early passages. Green fluorescence disappeared in all the iPSC colonies (Figure S1E). Importantly, SeV mRNA was not detected in iPSCs derived from PB-MNCs in late passages (Figure S1E).

In addition, to check whether the established protocol did allow preferential reprogramming in myeloid and/or progenitor cells, T cell receptor (TCR) and immunoglobulin heavy-chain genome rearrangements were studied on the iPSC generated (Figure S2). None of the analyzed iPSC clones (PB2iPSC c33, PKD2iPSC c78, PKD3iPSC c14, PKD3iPSC c10, and PKD3iPSC c35) had any T or B rearrangements, meaning that iPSC clones were generated from neither T nor B lymphocytes. These results guarantee the SeV-based reprograming system as the best option in reprogramming peripheral blood, as the reprograming vectors are cleared after iPSC generation, and the iPSC are generated from non-lymphoid cells. To continue with the following gene-editing steps clones from PB2, PKD2, and PKD3, we randomly selected different PB-MNC iPSC clones.

### TALEN-Based Gene Editing in the PKLR Locus of PKDiPSCs

**Figure 2:**
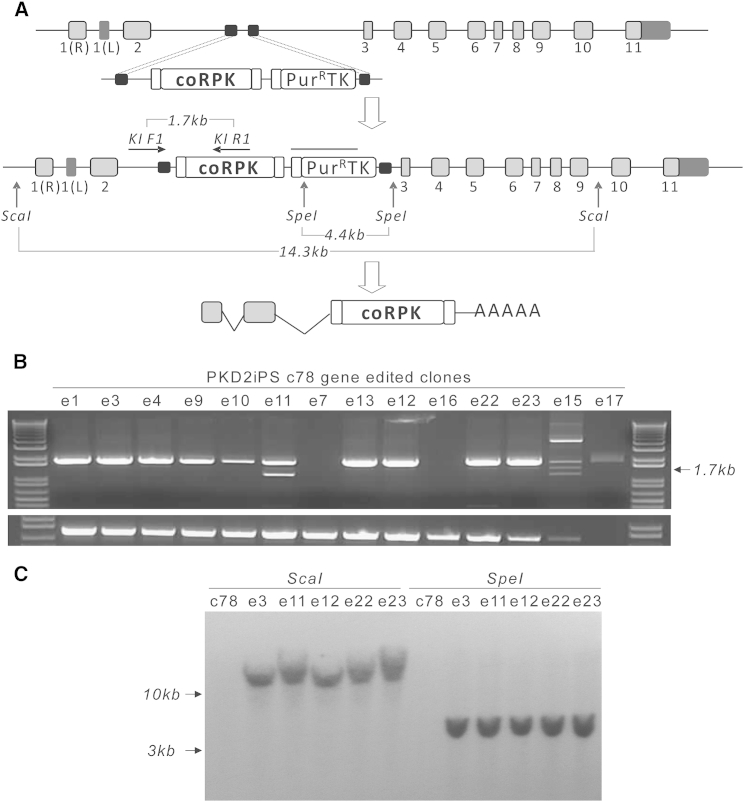
Gene Editing in the PKLR Locus (A) Diagram showing where therapeutic matrix is introduced by HR in the *PKLR* locus. The strategy to identify the integrated matrix by PCR (horizontal arrows) and Southern blot (vertical arrows) indicating the expected DNA fragment sizes is shown, and the line over the Puro^R^/thymidine kinase fusion cassette indicates probe location. Small squares at the beginning and end of the partial codon-optimized (cDNA)*RPK* indicate splicing acceptor and FLAG tag sequences present in the cassette, respectively; light gray squares represent endogenous (mRNA)*RPK* exons; dark gray squares represent the first LPK exon and 3′ UTRs at the beginning and at the end of the PKLR gene, respectively; and black squares represent homology arms. (B) DNA electrophoresis of gDNA from Puro^R^-PKD2iPSC clones, amplified by PCR to identify specific matrix integration. (C) Southern blot of gDNA from edited PKD2iPSC clones, digested by ScaI or SpeI to confirm the precise integration of the matrix in the *PKLR* locus. See also Figures S3 and S4 and Table S2.

To achieve correction of PKDiPSCs, we used a knockin gene-editing strategy based on inserting a therapeutic matrix containing a partial codon-optimized (cDNA)*RPK* gene covering exons 3 to 11, fused to a FLAG tag and preceded by a splice acceptor signal. Additionally, a positive-negative selection cassette containing a puromycin (Puro) resistance/thymidine kinase (TK) fusion gene driven by mouse phosphoglycerate kinase (mPGK) promoter was included downstream of the partial (cDNA)*RPK*. These elements were flanked by two homology arms matching sequences in the second intron of the *PKLR* gene (Figure 2A). In order to increase the efficiency of gene editing, we developed a *PKLR*-specific TALEN targeting a specific genomic sequence in the second intron flanked by the homology arms. Nuclease activity of the PKLR TALEN in the target sequence was verified by surveyor assay after nucleofecting both subunits of the nuclease in PKD2iPSC and PKD3iPSC (data not shown).

In two independent experiments, two iPSC lines from two different PKD patients, PKD2iPSC c78 and PKD3iPSC c54, were nucleofected with a control plasmid or with the developed matrix (from now on called therapeutic matrix) alone or together with two different doses of PKLR TALEN (1.5 or 5 μg of each PKLR TALEN subunit). Two days later, Puro was added to the media for 1 week. Puro-resistant (Puro^R^) colonies, with a satisfactory morphology appeared and were individually picked and subcloned. Most of the Puro^R^ colonies were identified from cells nucleofected with both the matrix and the PKLR TALEN subunits, although some colonies grew out after receiving only the therapeutic matrix. There was no difference in the number of Puro^R^ colonies between PKDiPSC lines from the different patients. To confirm target insertion of the therapeutic matrix in the second intron of the *PKLR* gene, we performed specific PCR analyses (Figures 2A and S3). The expected PCR product was detected in 10 out of 14 Puro^R^ clones from PKD2iPSC c78 and 31 out of 40 Puro^R^ clones from PKD3iPSC c54 (Figures 2B and S3). Taken together, we estimated an HR frequency among the Puro^R^ clones of above 75% for the two reprogramed patients (Table 1). In addition, two Puro^R^ clones from PKD3iPSC c54 clone nucleofected with the therapeutic matrix alone were positive for knockin, estimating an efficiency of 0.6 edited per 1 × 10^5^ nucleofected cells. Despite detecting HR without nucleases, the HR frequency was boosted almost five times (2.85 edited PKD3iPSC per 1 × 10^5^ nucleofected cells) when the PKLR TALEN was added. Additionally, knockin insertion of the therapeutic matrix was verified by Southern blot (Figure 2C), confirming a single insertion in the desired genomic locus.

Next, we tested whether the PKLR TALEN was also cutting the untargeted allele. Up to 40% of PKD2 and 31% of PKD3 edited clones carried insertions-deletions (indels) in the untargeted allele of the PKLR TALEN target site (Table 1; Figure S3C), demonstrating the high efficacy of this PKLR TALEN. Moreover, 3 out of 40 edited clones from PKD3iPSC were targeted biallelically as determined when both the targeted allele and the untargeted were analyzed in a single PCR (Figure S3D). In contrast, no edited PKD2iPSC clones showed biallelic targeting.

In order to check the specificity of the PKLR TALEN, we looked for potential off-target cutting sites in the different edited PKDiPSC clones. By in silico studies, we found five hypothetical off-target sites for this TALEN (Table S2). These five off-targets can be recognized by the two subunits matched as homodimers or heterodimer, where the left subunit can join the right subunit or each subunit can join a different spacer sequence and length (Table S2). All the potential off-targets had at least five mismatched bases, which makes the recognition by the TALEN unlikely. To confirm the specificity of the TALEN, we amplified genomic DNA from several edited PKD2iPSC and PKD3iPSC clones and Sanger sequenced around four off-targets (off-targets 1, 2, 4, and 5; data not shown). None of the analyzed clones showed any indels in any of the off-targets analyzed. Off-target 3 could not be amplified by PCR. Nevertheless, as the first base in the 5′ recognition site of the off-target 3 was an A, the recognition of this off-target by the PKLR TALEN is strongly reduced (Boch et al., 2009). This high specificity together with the high efficacy of PKLR TALEN confirms the feasibility of the developed TALEN and therapeutic matrix to promote HR in the *PKLR* locus.

Finally, we verified the pluripotency of the edited iPSCs after gene editing by in vivo teratoma formation into NSG mice (Figure S4). Edited clones were able to generate teratomas with tissues from the three embryonic layers. More importantly, human hematopoiesis, demonstrated by the presence of cells expressing the human CD45 panleukocytary marker (4.54% of the total teratoma forming cells) and human progenitors (CD45^+^CD34^+^; 2.74% of the total hCD45^+^ cells) derived from edited PKD3iPSC e31 teratomas could also be detected in vivo (Figure S4B). Altogether, the data confirm the use of PKLR TALEN to edit the *PKLR* gene in PKDiPSCs without affecting their pluripotent properties.

### A Single-Nucleotide Polymorphism Leads to Allele-Specific Targeting

**Figure 3:**
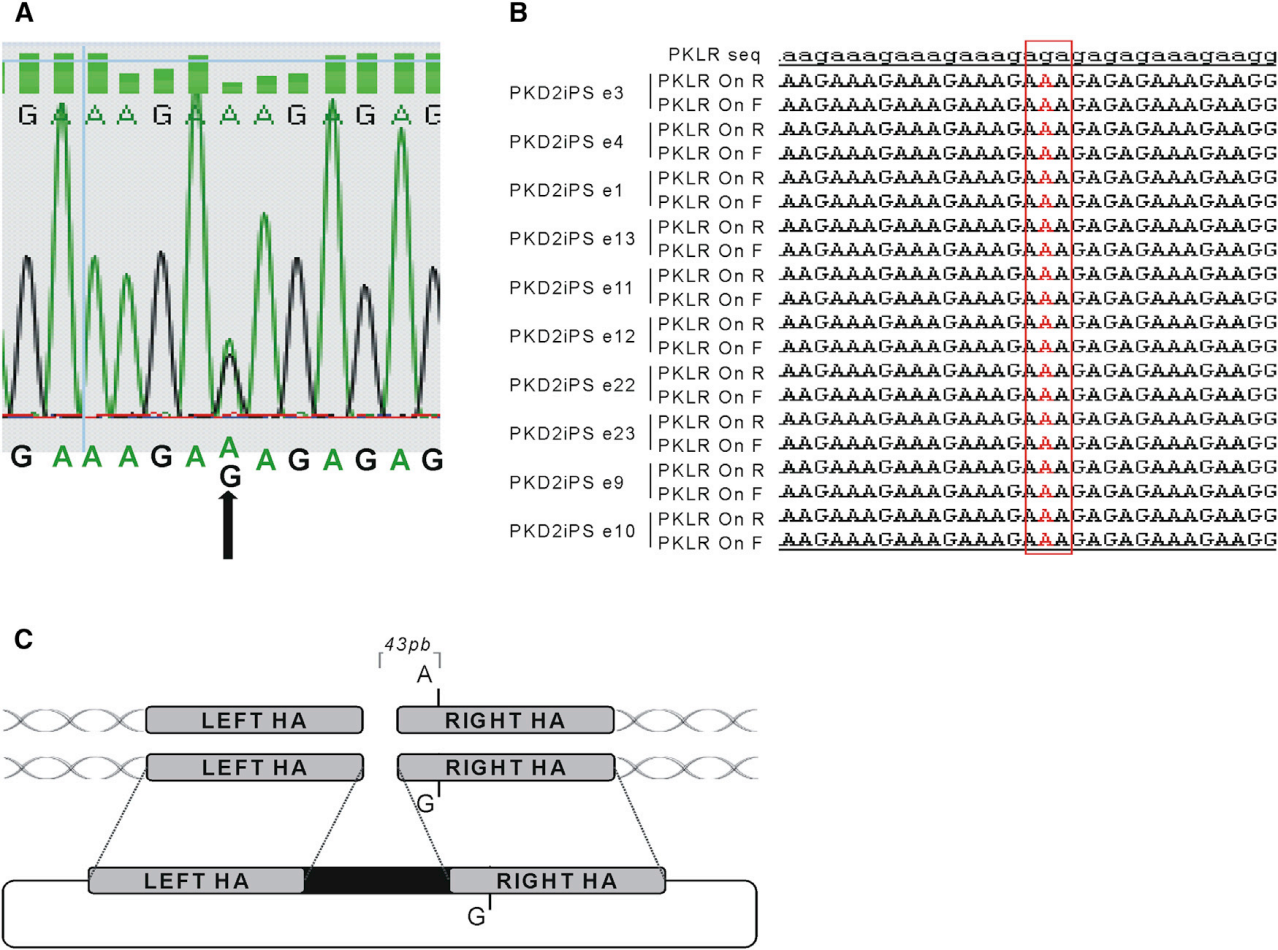
Allele-Specific Targeting on the PKLR Locus (A) A single-nucleotide polymorphism (SNP) detected in the second intron of the PKLR gene in PKD2 patient cells, identified by Sanger sequencing. Black arrow points to the polymorphism. (B) Sequence of PKD2 SNP in the untargeted allele in all the edited PKD2iPSC clones. Letter in red indicates the SNP. (C) Diagram indicating the position of the SNP with respect to the theoretical cutting site of the PKLR TALEN and the matrix integration in the targeted allele.

While evaluating the presence of indels in the untargeted allele by Sanger sequencing, we identified the existence of a g.[2268A > G] SNP 43 bases apart from the PKLR TALEN cutting site in PKD2iPSC (Figure 3A). Interestingly, the untargeted allele from all the edited PKD2iPSC clones (ten out of ten) carried the previously mentioned SNP, suggesting an impediment of the allele carrying the SNP variant to carry out HR. Moreover, no biallelic targeting was detected in any PKD2iPSC edited clone. On the contrary, 3 out of 31 edited PKD3iPSC clones without any SNP in the homology genomic area were targeted in both alleles.

### Genetic Stability of PKDiPSCs and Gene-Edited PKDiPSCs

We wanted to study whether the whole process of reprogramming plus gene editing was inducing genetic instability in the resulting cells. As a first approach, we performed karyotyping of the different iPSC lines and confirmed normal karyotype in all cases (data not shown). However, to have a clearer assessment, we monitored the genetic stability throughout all the process, including iPSC generation and gene-editing correction, by comparative genomic hybridization (CGH) and exome sequencing. PB-MNCs from a PKD2 patient, reprogrammed PKD2iPSC c58, and edited PKD2iPSC e11 were selected as representatives of each step. Copy-number variations (CNVs) were defined in these samples after comparing with a reference genomic DNA. Among the total CNVs identified, 31 were present in the original PB-MNC from PKD2, 34 CNVs were detected in PKD2iPSC c78, and 32 in PKD2iPSC e11 (Table 2; Table S3). Twenty-three CNVs detected in PKD2iPSC c78 were already present in PKD2 PB-MNCs, indicating the mosaicism of the original patient sample. On the other hand, only four CNVs present in PKD2iPSC c78 and PKD2iPSC e11 were not detected in the primary sample. Of note, these four CNV were at chromosomes 1q44, 2p21, 3p12.3-p12.1, and Xp11.22, involving genes such as *ROBO1*, *GBE1*, *TCEA1*, *LYPLA1*, *DLG2*, *PLEKHA5*, and *AEBP2* (Table 2). More importantly, only two CNVs appeared after gene editing that were not present in the original iPSC clone. The first one was a deletion of 6.6 kb that include several olfactory receptor genes (such as *OR2T11*, *OR2T35*, or *OR2T27*), and the second CNV was an amplification of 0.6 kb that includes the *FGD1* gene. Additionally, sequences surrounding these two CNVs in PKD2iPSC e11 have more than eight mismatches with the PKLR TALEN recognition site, suggesting that these genomic alterations were not produced by gene editing. Moreover, we analyzed the presence of CNVs in PKD3iPSC before and after gene editing to confirm the potential harmless effect in the genomic stability of PKLR TALEN activity (Table S4). Edited clone PKD3iPSC e31 (biallelically targeted) showed 10 out 11 CNVs of the parental PKD3iPSC c54, and PKD3iPSC e88 (monoallelically targeted) showed two new CNVs. Furthermore, none of the CNVs present in the edited PKD2iPSC e11 were present in any of these two PKD3iPSC edited clones, which suggests that PKLR TALEN does not induce any specific CNVs in PKDiPSC clones.

Simultaneously, the three PKD2 samples were assayed using the Illumina HiSeq 2000 system for exome sequencing. After bioinformatics analysis by comparing the sequencing data with a human genome reference, PKD2 PB-MNCs showed 68,260 changes in their sequences, PKD2iPSC c78 68,542, and PKD2iPSC e11 67,728 (Table S5). Only ten of all variants detected in PKD2iPSC e11 were in exonic regions, included in the SNP database, and not identified in PKD2 PB-MNCs (Table 2). Additionally, four of them were also detected in PKD2iPSC c78. In order to verify the presence of these mutations by Sanger sequencing, we PCR amplified and sequenced these regions. Only the mutations in the *RUSC2*, *TACR2*, and in *APOA5* genes could be confirmed by sequencing (data not shown). None of the ten variants were included in the COSMIC database (Wellcome Trust Sanger Institute, 2014), which includes all the known somatic mutations involved in cancer.

Overall, genetic stability analysis confirmed the safety of our gene editing approach. All the genetic alterations identified were present in the PB-MNCs or generated during their reprogramming or iPSC expansion. Moreover, none of the confirmed alterations could be associated with potentially dangerous mutations.

### Gene-Edited PKDiPSCs Recover RPK Functionality

**Figure 4:**
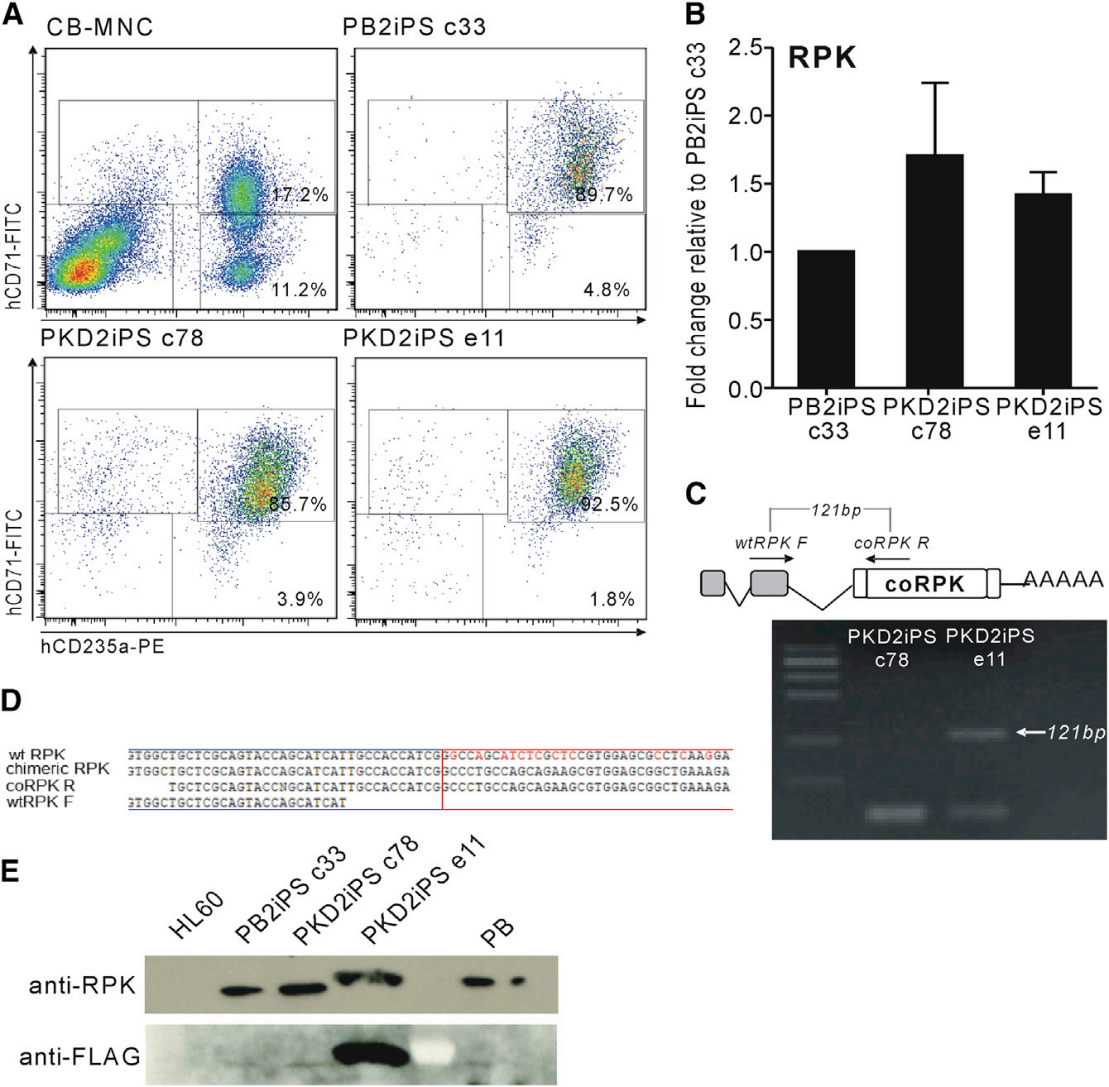
Erythroid Differentiation of PKD2iPSCs PB2iPSCs, PKD2iPSCs, and edited PKD2iPSCs were differentiated to erythroid cells under specific conditions and analyzed after 31 days in in vitro proliferation and differentiation conditions. (A) Erythroid differentiation was confirmed by flow cytometry analysis. Cord blood MNCs, PB2iPSC clone c33, PKD2iPC clone c78, and edited PKD2iPSC clone e11 representative analyses are shown. (B) RPK expression in erythroid cells derived from the different iPSCs was evaluated by qRT-PCR (n = 6). (C) Specific RT-PCR to amplify the chimeric (mRNA)*RPK* in edited PKD2iPSC. The primers amplified the region around the link between endogenous (mRNA)*RPK* and the introduced codon-optimized (cDNA)*RPK* sequence. Arrow indicates the expected band and the corresponding size only preset in the RNA from edited cells (PKD2iPSC e11). (D) The sequence of the chimeric transcript was aligned with the theoretical expected sequence after the correct splicing between the endogenous exon 2 (blue square) and the exogenous exon 3 (red square). (E) The presences of RPK protein in erythroid cells derived from PB2iPSCs, PKD2iPSCs, and edited PKD2iPSCs assessed by western blot (upper line); mobility change in PKD2iPSC e11 is due to the FLAG tag added to the chimeric protein. Expression of chimeric protein was detected by anti-FLAG antibody only in erythroid cells derived from edited PKD2iPSCs (bottom line). See also Figures S5 and S6.

Once the knockin integration was confirmed, we assessed the PK phenotypic correction of the gene-edited iPSCs. We induced the erythroid differentiation of different iPSC lines from a healthy donor iPSC line (PB2iPSC c33), PK-deficient iPSC lines derived from both patients (PKD2iPSC c78 and PKD3iPSC c54), and the corresponding edited clones (monoallelically edited PKD2iPSC e11 and PKD3iPSC e88 and a biallelically targeted PKD3iPSC e31). Characteristic hematopoietic progenitor markers, such as CD43, CD34, and CD45, started to appear over time (data not shown) and were expressed in a similar proportion of cells derived from all of the iPSC lines. Erythroid cells were clearly observed in the cultures (Figure S5A), and the specific erythroid combination of CD71 and CD235a antigens was expressed on the majority of cells after 21 days of differentiation (Figures 4A and S5B). Moreover, cells derived from all iPSC lines analyzed at day 31 of differentiation, showed a similar globin pattern, in which α- and γ-globins were predominant with a small amount of β-globin, and residual embryonic ε- and ζ-globins detected, confirming the erythroid differentiation of these pluripotent lines (Figure S6A). More importantly, the erythroid cells derived from the three iPSC lines were able to express RPK (Figures 4B, 4E, S5C, and S5F). It is noteworthy that no alteration in the expression of proximal genes in the edited erythroid cells was confirmed by qRT-PCR (Figure S6B).

The presence of chimeric transcripts in all of the edited PKDiPSC lines was confirmed by RT-PCR. Primers recognizing a sequence in the second endogenous exon of the *PKLR* gene and in the partial codon-optimized (cDNA)*RPK* were able to produce an amplicon with the correct size, specifically in erythroid cells derived from gene-edited PKDiPSCs (Figures 4C and S5E). This amplicon was sequenced and the joint between both parts of the mRNA, coming from the transcription of the endogenous and the exogenous sequences, was detected (Figure 4D). Additionally, the presence of RPK was demonstrated by western blot in the erythroid cells derived from all of the edited iPSC lines derived from PKD2iPSC c78 (PKD2iPSC e11; Figure 4E) and from PKD3iPSC c54 (PKD3iPSC e88 and PKD3iPSC e31; Figure S5E). Interestingly, although (mRNA)*RPK* could be detected in erythroid cells derived from all the iPSC lines derived from PKD3 (Figure S5C), RPK protein was not detected in PKD3iPSC c54 (Figure S6F), probably due to the severity of the mutation in terms of RNA translation. However, the gene edition of PKD3iPSC restored RPK protein expression either in the bialellic (PKD3iPSC e31) and monoallelic (PKD3iPSC e88) edited lines (Figure S5F). Moreover, both the level of the chimeric transcript and the RPK protein were higher in the biallelically targeted clone PKD3iPSC e31 than in the monoallelic PKD3iPSC e88 (Figures S5D and S5F). It is worth it mentioning that flagged RPK was detected in erythroid cells generated after gene editing of PKDiPSCs (Figure 4E), confirming the origin of the RPK protein from the edited genome.

**Figure 5:**
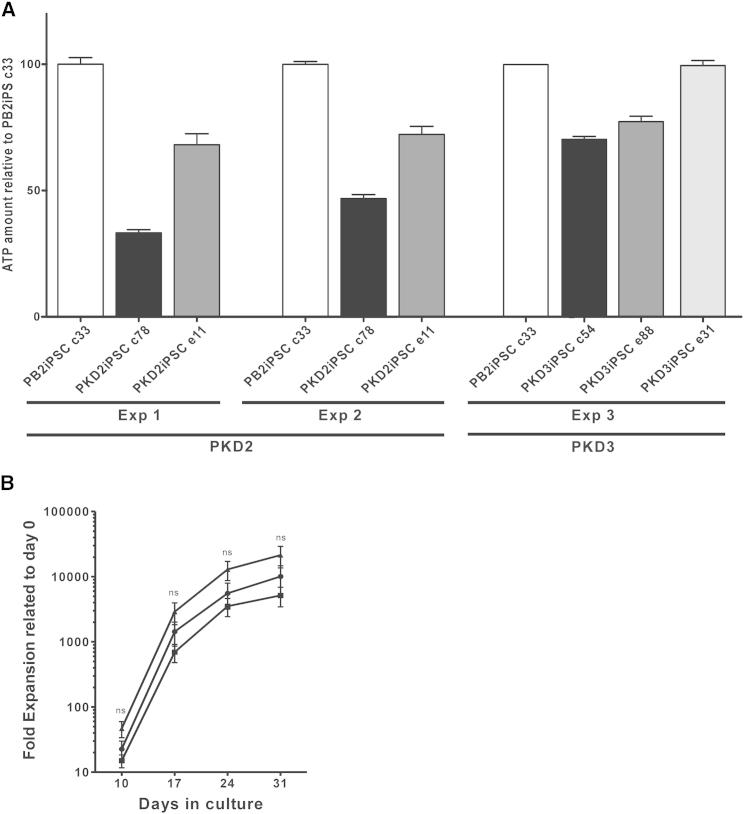
Phenotypic Correction in Edited PKD2iPSCs (A) ATP levels in erythroid cells derived from healthy iPSCs (PB2iPSCs), PKDiPSCs (patients PKD2 and PKD3), and edited PKDiPSCs (PKD2iPSC e11, PKD3iPSC e88, and PKD3iPSC e31 clones). Data were obtained from three independent experiments from six different iPSC lines derived from two different patients. (B) In vitro proliferation and differentiation of PB2iPSC clone c33 (▪), PKD2iPC clone c78 (▲), and edited PKD2iPSC clone e11 (●). ns, statistically not significant. See also Figure S7.

Finally, the recovery in metabolic function of the corrected cells was assessed in the differentiated cells by conventional biochemical analysis as well as by liquid chromatography mass spectrometry (LC-MS) (Figures 5 and S7). The ATP level in erythroid cells derived from the monoallelically edited PKDiPSCs (PKD2iPSC e11 and PKD3iPSC e88) was augmented after gene editing (Figure 5A), reaching an intermediate level between that observed in erythroid cells from WT iPSCs and their respective patient-specific iPSC lines. Additionally, erythroid cells derived from the biallelically targeted PKD3iPSC e31 restored the ATP level completely up to healthy values (Figure 5A). In edited erythroid cells, other glycolytic metabolites, such as 2,3-diphosphoglyceric acid, 2-phosphoglyceric acid, pyruvic acid, and L-lactic acid, reached levels between those of control and deficient erythroid cells derived from PB2iPSCs and PKDiPSCs (Figure S7). In addition, we obtained up to 2 × 10^4^-fold expansion of cells in 1 month, meaning that up to 20,000 erythroid cells could be generated from a single iPSC (Figure 5B). As expected, no statistical differences were observed between the different iPSCs, indicating that RPK deficiency only affects the last steps of the erythroid differentiation, where no proliferation is taking place. Altogether, our data validate the effectiveness of this knockin approach to express a corrected RPK protein and demonstrate its potential to therapeutically correct the PKD phenotype and generate large numbers (10^9^–10^10^) of differentiating cells required for comprehensive biochemical and metabolic analyses during their maturation, or even for a potential therapeutic use.

## Discussion

In this work, we have shown the potential to combine cell reprograming and gene editing as a therapeutic approach for PKD patients. We generated iPSCs from PB-MNCs taken from PKD patients using a non-integrating viral system. These PKDiPSC lines were effectively gene edited via a knockin strategy at the *PKLR* locus, facilitated by specific PKLR TALENs. More importantly, we have demonstrated the rescue of the disease phenotype in erythroid cells derived from edited PKDiPSCs by the partial restoration of the step of the glycolysis affected in PKD and the improvement of the total ATP level in the erythroid cells derived from PKDiPSCs. The restoration of the energetic balance in erythroid cells derived from PKD patients opens up the possibility of using gene editing to treat PKD patients.

To reprogram patient cells, we adopted the most feasible and safest protocol using a patient cell source that is easy to obtain, PB-MNCs, and an integration-free reprogramming strategy based on SeV vectors. PB-MNCs were chosen, as blood collection is common in patient follow-up and is minimally invasive. Additionally, it is possible to recover enough PB-MNCs from a routine blood collection to perform several reprogramming experiments. Finally, previous works showed that PB-MNCs could be reprogrammed, although at a very low efficiency (Staerk et al., 2010). On the other hand, the SeV reprogramming platform has been described as a very effective, non-integrative system for iPSC reprogramming with a wide tropism for the target cells (Ban et al., 2011, Fusaki et al., 2009). Reprogrammed SeVs are cleared after cell reprogramming due to the difference of replication between newly generated iPSCs and viral mRNA (Ban et al., 2011, Fusaki et al., 2009). However, reprogrammed T or B cells might be favored when whole PB-MNCs are chosen, as these are the most abundant nucleated cell type in these samples. Reprogramming T or B cells has the risk of generating iPSCs with either TCR or immunoglobulin rearrangements, decreasing the immunological repertoire of the hematopoietic cells derived from these rearranged iPSCs. In order to avoid this possibility, we have biased the protocol against reprogramming of either T or B lymphocytes by culturing PB-MNCs with essential cytokines to favor the maintenance and proliferation of hematopoietic progenitors and myeloid cells, as previously shown for retroviral reprogramming vectors (Staerk et al., 2010). This approach was supported here by the demonstration that SeV vectors preferentially transduced hematopoietic progenitors and myeloid cells under these specific conditions and consequently none of the iPSC lines analyzed had immunoglobulin or TCR rearrangements. We further demonstrated that the generation of iPSCs from PB-MNCs using SeV is feasible and simple and generates integration-free iPSC lines with all the characteristic features of true iPSCs that could be further used for research or clinical purposes.

The next goal for gene therapy is the directed insertion of the therapeutic sequences in the cell genome (Garate et al., 2013, Genovese et al., 2014, Karakikes et al., 2015, Song et al., 2015). A number of different gene-editing strategies have been described, including gene modification of the specific mutation, integration of the therapeutic sequences in a safe harbor site, or knockin into the same gene locus. We directed a knockin strategy to insert the partial cDNA of a codon-optimized version of *RPK* in the second intron of the PKLR gene. If used clinically, this strategy would allow the treatment of up to 95% of the patients, those with mutations from the third exon to the end of the (cDNA)*RPK* (Beutler and Gelbart, 2000, Fermo et al., 2005, Zanella et al., 2005). Additionally, this approach retained the endogenous regulation of RPK after gene editing, a necessary factor as RPK is tightly regulated throughout the erythroid differentiation. This fine control would be lost if a safe-harbor strategy was chosen.

The PKLR TALEN generated was very specific and very efficient. We did not find any mutation in any of the theoretical off-target sites defined by the off-site search algorithm and analyzed by PCR and gene sequenced. Moreover, we determined that 2.85 out to 100,000 electroporated PKDiPSCs, without considering the toxicity associated to nucleofection, were gene edited when the PKLR TALEN was used, reaching values similar to those previously published by others (Porteus and Carroll, 2005). Interestingly, 40% of the edited PKDiPSC clones presented indels in the untargeted allele or were biallelically targeted, which indicated that the developed TALEN are very efficient, cutting on the on-target sequence with a high frequency.

Surprisingly, we found that the presence of a single SNP 43 bp away from the PKLR TALEN cutting site was an impediment to HR. The presence of an SNP, which disrupts the complete matching between the genome sequence and homology arm, has already been reported to reduce the frequency of HR (Deyle et al., 2014). Taking into account that the TALEN cut has occurred, as we can detect indels in the non-targeted allele, the absence of matrix insertion seems to be directly related to problems related with the perfect annealing of the matrix with the genome sequences. We have to point out that this SNP is located in a very repetitive region, which might form a structural configuration that increases the HR specificity between this region and its homology arm, as has already been mentioned (Renkawitz et al., 2014). Thus, the genome context where the HR has to take place plays an important role and can facilitate or impair HR. In any case, these data demonstrate the important need for gene-editing strategies to generate the homology arms of an HR matrix from the individual DNA that will be edited. This would restrict HR matrices to patients with similar SNPs in the genomic region to be edited. Therefore, any gene-editing therapy using a knockin or safe-harbor strategy should first screen each patient for the presence of an SNP in the homology arms selected. On the other hand, the presence of a specific SNP could also help to perform allele-specific gene targeting in the cases where the presence of a dominant allele is pathogenic as, for example, in α-thalassemia (De Gobbi et al., 2006).

The gene-editing strategy utilized here to correct PKD was safe, since neither the introduction of genomic alterations nor alteration of the expression of neighboring genes by the insertion and expression of the exogenous sequences occurred. This demonstrates the safety of this knockin gene-editing strategy without *cis* activation of any gene, in comparison to previous results where the selection cassette deregulated nearby genes (Zou et al., 2011). Furthermore, we did not observe any off-target effects induced by PKLR TALEN gene editing.

We found several genomic alterations by CGH and exome sequencing analysis. However, the majority of them were already present in PKD PB-MNCs before their reprogramming, especially in the case of the biallelic targeted PKD3iPSC c31 (Table S4), where all of the CNVs were already present in PKD3iPSC c54, confirming previous data associating these DNA variations in iPSC clones with a cellular mosaicism in the original samples (Abyzov et al., 2012). However, there were some mutations present in the iPSC that we were unable to detect in the original sample, which might be due to technical limitations or to the inherent genetic instability associated with the reprogramming process and iPSC culture (Gore et al., 2011, Hussein et al., 2011). Supporting this last possibility, we found CNVs present in PKD2iPSC c78 and not in PKD2iPSC e11 (Table 2; Table S3). Because PKD2iPSC c78 was maintained in vitro for several more passages, after HR and before CGH analysis, some new changes could have occurred that were not present in the gene-edited-derived clones. Although one CNV involved the *TCEA1* gene, indirectly involved in salivary adenoma as a translocation partner of *PLAG1* (Asp et al., 2006), none of these genomic alterations identified were implicated in hematopoietic malignancies, cell proliferation, or apoptosis regulation, suggesting their neutrality in the PKD therapy by gene editing.

Constitutive expression of Puro/TK from the ubiquitously active mPGK promoter might hinder therapeutic applications of this approach. Indeed, these highly immunogenic prokaryotic/viral proteins can be presented on the cell surface of the gene-corrected cells by the major histocompatibility complex class I molecules, thus stimulating an immune response against the cells once transplanted into the patients. Here, although the Puro/TK cassette has been maintained in the edited PKDiPSC lines, the cassette is inserted between two *loxP* sites, which would allow us to excise it before their clinical application. Moreover, for the potential clinical use of our approach, other selection systems could be used, such as a truncated version of the nerve growth factor receptor combined with enrichment by magnetic sorting, or the use of an inducible or an embryonic-specific promoter instead of the PGK constitutive promoter to limit the Puro/TK expression.

Finally, we have clearly demonstrated the effectiveness of editing the *PKLR* gene in PKDiPSCs to recover the energetic balance in erythroid cells derived from edited PKDiPSCs. ATP and other metabolites involved in glycolysis were restored by expressing a chimeric RPK in a physiological manner. As expected erythroid cells derived from monoallelic corrected PKDiPSCs produce partial restoration of ATP levels, and erythroid cells derived from biallelic corrected PKD3iPSC e31 fully recovered ATP level (Figure 5A). Additionally, we could not observe any difference in the erythroid populations obtained in vitro from uncorrected and corrected PKDiPSCs, probably due to the lack of terminal differentiation/enucleation of the protocol used to generate mature enucleated erythrocytes. Furthermore, we were able to generate 20,000 erythroid cells per starting iPSC, providing abundant material for our assays and offering the potential to undertake more comprehensive analyses, including metabolic and biochemical profiling, to further elucidate the effects of PKD on erythroid cells, or even for therapeutic usage.

Many groups are working to generate long-term reconstituting HSCs from iPSCs, and a major development was reported by Amabile et al. (2013), who showed that in vivo differentiation of human iPSCs in NSG mice reveals their intrinsic potential to fully reconstitute the hematopoietic system. We confirmed the in vivo hematopoietic potential of gene-edited PKDiPSCs (even hematopoietic progenitors could be detected), but we failed to generate in vivo engraftable hematopoietic progenitors (data no shown), possibly because of the low efficacy of our in vivo hematopoietic differentiation approach, which we are working to improve.

In summary, we combined gene editing and patient-specific iPSCs to correct PKD. Our gene-editing strategy was based on inserting a partial codon-optimized (cDNA)*RPK* in the *PKLR* locus mediated by PKLR TALEN without altering the cellular genome or neighbor gene expression. Additionally, we found a highly homologous sequence specificity, since a single SNP could avoid HR. The resultant edited PKDiPSC lines could be differentiated to large number of erythroid cells, where the energetic defect of PKD erythrocytes was effectively corrected. This validates the use of iPSCs for disease modeling and demonstrates the potential future use of gene editing to correct PKD and also other metabolic red blood cell diseases in which a continuous source of fully functional erythrocytes is required.

## Experimental Procedures

### Peripheral Blood Samples and Reprogramming

Peripheral blood from PKD patients and healthy donors was collected in routine blood sampling from Hospital Clínico Infantil Universitario Niño Jesús (Madrid, Spain), Centro Hospitalario de Coimbra (Coimbra, Portugal), and the Medical Care Service of CIEMAT (Madrid, Spain). All samples were collected under written consent and institutional review board agreement. PB-MNCs were isolated by density gradient using Ficoll-Paque (GE Healthcare). PB-MNCs were pre-stimulated for 4 days in StemSpan (STEMCELL Technologies) plus 100 ng/ml human stem cell factor (SCF), 100 ng/ml hFLT3L, 20 ng/ml hTPO, 10 ng/ml G-CSF, and 2 ng/ml human IL-3 (Peprotech) (Figure 1A). Cells were then transduced with a mix of SeV, kindly provided by DNAvec (Japan), expressing OCT3/4, KLF4, SOX2, c-MYC, and Azami Green, each at a MOI of 3. Transduced cells were maintained for four more days in the same culture medium and then supplemented with 10 ng/ml basic fibroblast growth factor (FGF). Five days after transduction, cells were collected and seeded on irradiated human foreskin fibroblast (HFF-1)-coated (ATCC) culture plates with human ES media (knockout DMEM, 20% knockout serum replacement, 1 mM L-glutamine, and 1% nonessential amino acids [all from Life Technologies]), 0.1 mM β-mercaptoethanol (Sigma-Aldrich), and 10 ng/ml basic human FGF (Peprotech). Human ES media was changed every other day. When human ES-like colonies appeared, they were selected under the stereoscope (Olympus) and a clonal culture from each colony was established.

### Gene Editing in iPSCs

iPSCs were treated with Rock inhibitor Y-27632 (Sigma) before a single-cell suspension of iPSCs was generated by StemPro Accutase (Life Technologies) treatment and then nucleofected with 1.5 μg or 5 μg of each PKLR TALEN subunit with or without 4 μg HR matrix by Amaxa Nucleofector (Lonza) using the A23 program. After nucleofection, cells were seeded into a feeder of irradiated Puro^R^ mouse embryonic fibroblasts in the presence of Y-27632, and 48 hr after transfection, puromycin (0.5 μg/ml) was added to human ES media. Newly formed Puro^R^-PKDiPSC colonies were picked individually during a puromycin selection period of 6–10 days. Puro^R^-PKDiPSC colonies were expanded and analyzed by PCR and Southern blot to detect HR (Figures 2B, 2C, S3B, and S3D).

### Erythroid Differentiation

Erythroid differentiation from iPSC lines was performed using a patented method (WO/2014/013255). In brief, we used a multistep, feeder-free protocol developed by E.O. (unpublished data). Before differentiation, normal, diseased, and corrected iPSCs were maintained in StemPro medium (Life Technologies) with the addition of 20 ng/ml basic FGF on a matrix of recombinant vitronectin fragments (Life Technologies) using manual passage. For initiation of differentiation, embryoid bodies (EBs) were formed in Stemline II medium (Sigma Aldrich) with BMP4, vascular endothelial growth factor (VEGF), Wnt3a, and activin A. In a second step, hematopoietic differentiation was induced by adding FGFa, SCF, IGF2, TPO, and heparin to the EB factors. After 10 days, hematopoietic progenitors were harvested and replated into fresh Stemline II medium supplemented with BMP4, SCF, Flt3 ligand, IL-3, IL-11, and erythropoietin (EPO) to direct differentiation along the erythroid lineage and to support extensive proliferation. After 17 days, cells were transferred into Stemline II medium containing a more specific erythroid cocktail that included insulin, transferrin, SCF, IGF1, IL-3, IL-11, and EPO for 7 days. In a final maturation step of 7 days (days 24–31), cells were transferred into IMDM with insulin, transferrin, and BSA and supplemented with EPO. Cells were harvested for analysis on days 10, 17, 24, and 31.

## Author Contributions

Z.G., O.Q.-B., A.M.C., E.O., C.H., X. A., I.O., L.C., O.A., F.R.-F., F.G.-S., G.G., and J.C.S. performed experiments. Z.G., O.Q.-B, E.O., P.K., C.H., C.K., X.A., F.G.-S., F.P., S.J., J.M., B.R.D., and J.C.S. analyzed results. L.P., R.G., N.F., T.M.M., M.L.R., J.S., and A.G. designed reagents and provided samples. Z.G., O.Q.-B., J.A.B, B.R.D., and J.C.S. designed research. O.Q.-B. and J.C.S. wrote the paper. J.C.S. obtained financial support.

## Figures and Tables

**Table 1:** Efficacy of Homologous Recombination in PKD2iPSCs and PKD3iPSCs and Indels Analysis in the Untargeted Allele

	Puro^R^ Clones	Percentage of Gene-Edited Clones	Percentage of Gene-Edited Clones Targeted Biallelically	Percentage of Gene-Edited Clones with Indels in the Untargeted Allele
PKD2iPSCs	13	77%	0%	40%
PKD3iPSCs	40	76%	11%	31%

**Table 2:** Copy-Number Variations and Exome Variants Detected by CGH and Exome Sequencing in Edited PKD2iPSCs

CGH Analysis						
**Number**	**Chromosome**	**Cytoband**	**Size (bp)**	**Type**	**Present in PKD2iPSC c78**	**Present in PKD2 PB-MNCs**

1	1	q44	60,641	DEL	no	no
2	3	p12.2-p12.1	3,931,633	LOH	yes	no
3	8	q11.23	169,460	AMP	yes	no
4	11	q14.1	113,264	DEL	yes	no
5	12	p12.3	1,182,747	AMP	yes	no
6	17	q21.31	199,747	AMP	yes	no
7	X	p11.22	6,030	AMP	no	no

**Exome Sequencing**						

**Number**	**Chromosome**	**Reference Base**	**Altered Base**	**Gene**	**Type**	**Present in PKD2iPSC c78**

1	9	−	TGCCTCCACCACACC	PHF2	nonframeshift insertion	no
2	16	G	T	ZNF747	nonsynonymous SNV	no
3	6	G	C	SNX3	nonsynonymous SNV	no
4	22	A	T	TUBGCP6	nonsynonymous SNV	no
5	10	A	G	TARC2	nonsynonymous SNV	no
6	7	C	A	TNRC18	stop-gain SNV	no
7	18	C	A	MBD2	nonsynonymous SNV	yes
8	18	C	A	MBD2	nonsynonymous SNV	yes
9	9	G	T	RUSC2	nonsynonymous SNV	yes
10	11	G	A	APOA5	nonsynonymous SNV	yes

SNV, single-nucleotide variation. See also Tables S4 and S5.

## Supplemental Information

Document S1. Supplemental Experimental Procedures, Figures S1–S7, and Tables S1, S2, and S4

Table S3. CNVs Identified in PKD2 PB-MNCs, PKD2iPSC c78, and PKD2iPSC e11, Related to Table 2

Table S5. Variants Identifying by Exome Sequencing in PKD2 PB-MNC, PKD2iPSC c78, and PKD2iPSC e11, Related to Table 2

Document S2. Article plus Supplemental Information
