# Supplementary material for: Generation of a High Number of Healthy Erythroid Cells from Gene-Edited Pyruvate Kinase Deficiency Patient-Specific Induced Pluripotent Stem Cells
Source: Stem Cell Reports. 2015 Nov 5;5(6):1053–66. doi: 10.1016/j.stemcr.2015.10.002 (PMC4682065; doi:10.1016/j.stemcr.2015.10.002)
Supplement: Document S1. Supplemental Experimental Procedures, Figures S1–S7, and Tables S1, S2, and S4 [file mmc1.pdf]

Stem Cell Reports

Supplemental Information

Generation of a High Number of Healthy Erythroid Cells from Gene-Edited Pyruvate Kinase Deficiency Patient-Specific Induced Pluripotent Stem Cells

Zita Garate, Oscar Quintana-Bustamante, Ana M. Crane, Emmanuel Olivier, Laurent Poirot, Roman Galetto, Penelope Kosinski, Collin Hill, Charles Kung, Xabi Agirre, Israel Orman, Laura Cerrato, Omaira Alberquilla, Fatima Rodriguez-Fornes, Noemi Fusaki, Felix Garcia-Sanchez, Tabita M. Maia, Maria L. Ribeiro, Julian Sevilla, Felipe Prosper, Shengfang Jin, Joanne Mountford, Guillermo Guenechea, Agnes Gouble, Juan A. Bueren, Brian R. Davis, and Jose C. Segovia

Figure S1

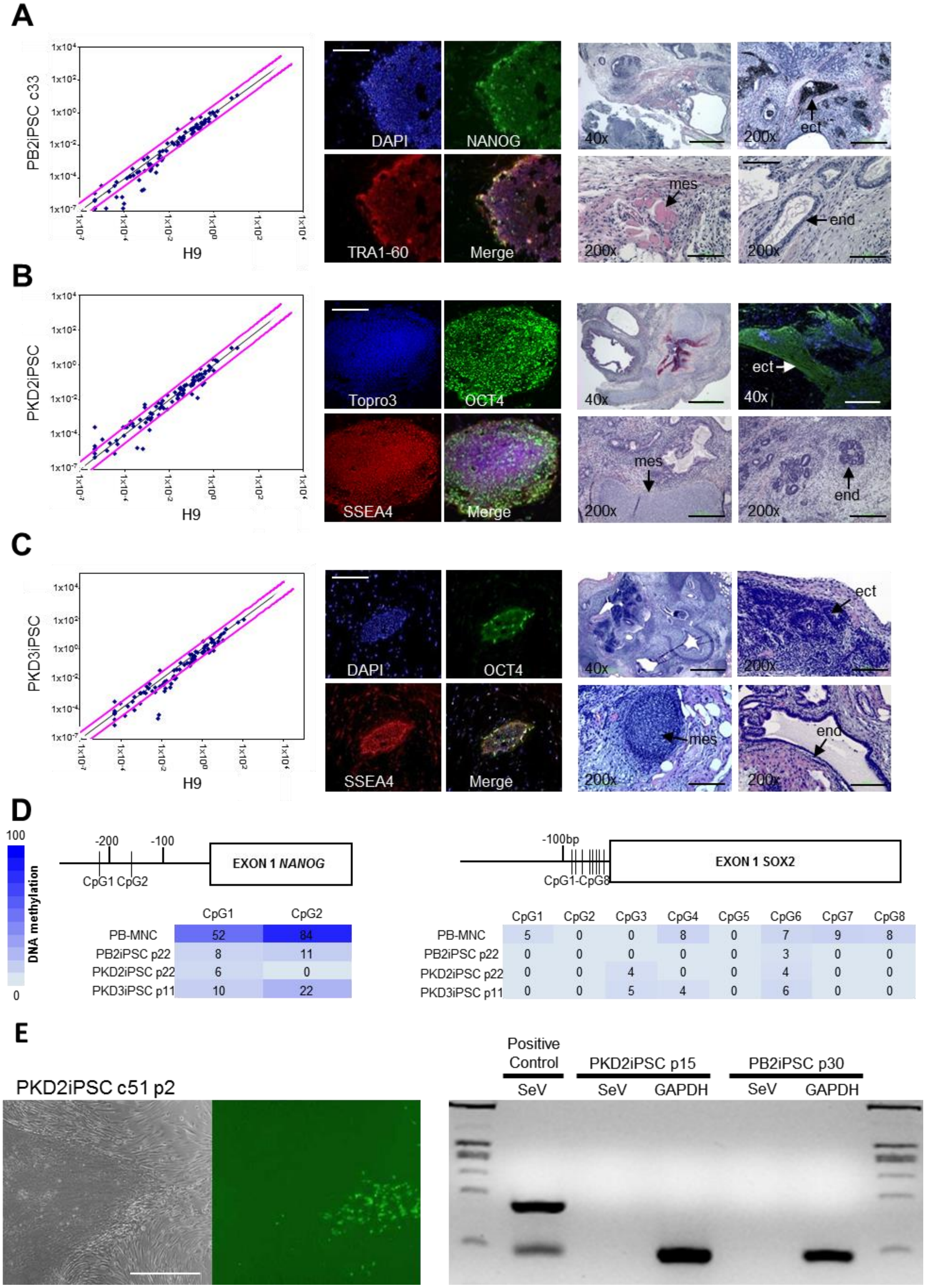

A
PB2iPSC c33
H9
DAPI
NANOG
TRA1-60
Merge
40x
200x
ect
mes
end
B
PKD2iPSC
H9
Topro3
OCT4
SSEA4
Merge
40x
200x
ect
mes
end
C
PKD3iPSC
H9
DAPI
OCT4
SSEA4
Merge
40x
200x
ect
mes
end
D
100
DNA methylation
0
-200
-100
CpG1 CpG2
EXON 1 NANOG
CpG1 CpG2
PB-MNC 52 84
PB2iPSC p22 8 11
PKD2iPSC p22 6 0
PKD3iPSC p11 10 22
-100bp
CpG1-CpG8
EXON 1 SOX2
CpG1 CpG2 CpG3 CpG4 CpG5 CpG6 CpG7 CpG8
PB-MNC 5 0 0 8 0 7 9 8
PB2iPSC p22 0 0 0 0 0 3 0 0
PKD2iPSC p22 0 0 4 0 0 4 0 0
PKD3iPSC p11 0 0 5 4 0 6 0 0
E
PKD2iPSC c51 p2
Positive Control
PKD2iPSC p15
PB2iPSC p30
SeV
SeV
GAPDH
SeV
GAPDH

Figure S1, related to Figure 1. Characterization of PB2iPSC and PKDiPSC lines. (**A-C**) RT-PCR stem array analysis (left), immunofluorescence analysis of hESC markers (middle)(scale bars 100μm) and representative photograph of different tissues from the three embryonic layers (right) (scale bars 200μm for 40x and 50μm for 200x) of (**A**) PB2iPSC, (**B**) PKD2iPSC and (**C**) PKD3iPSC. (**D**) Methylation on different CpGs islands in *NANOG* promoter (left) and *SOX2* promoter (right) assessed in control MNC, PB2iPSC, PKD2iPSC and PKD3iPSC by pyrosequencing. A reduction in the amount of methylation in the different iPSC lines is observed. (**E**) Absence of Azami green in iPSC colonies at passage 2 (left) (scale bar 50μm) and analysis of SeV genomic RNA in iPSC lines by RT-PCR at passages 15 and 30 (right).

Figure S2

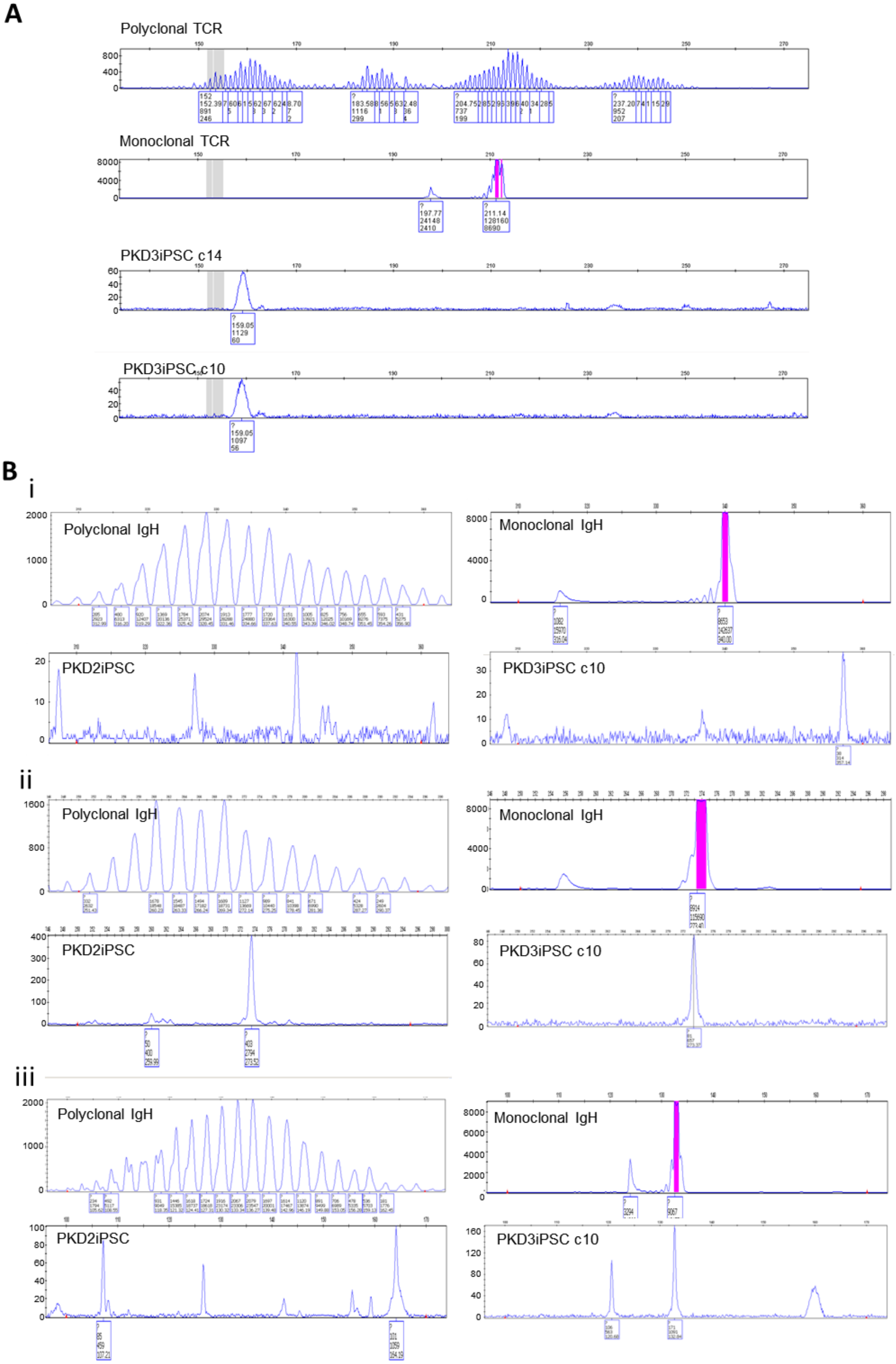
A
Polyclonal TCR
Monoclonal TCR
PKD3iPSC c14
PKD3iPSC c10
B
i
Polyclonal IgH
Monoclonal IgH
PKD2iPSC
PKD3iPSC c10
ii
Polyclonal IgH
Monoclonal IgH
PKD2iPSC
PKD3iPSC c10
iii
Polyclonal IgH
Monoclonal IgH
PKD2iPSC
PKD3iPSC c10

Figure S2, related to Figure 1. TCR and IgH genome rearrangement study. (**A**) TCR rearrangements in iPSC colonies. Polyclonal and monoclonal samples are included as reference. Examples of two iPSC clones are shown where only background is detected. (**B**) IgH rearrangements in iPSC colonies: (i) IGH FR1. (ii) IGH FR2. (iii) IGH FR3. A polyclonal sample is included as reference. Examples of two PKDiPSC clone are showed.

Figure S3

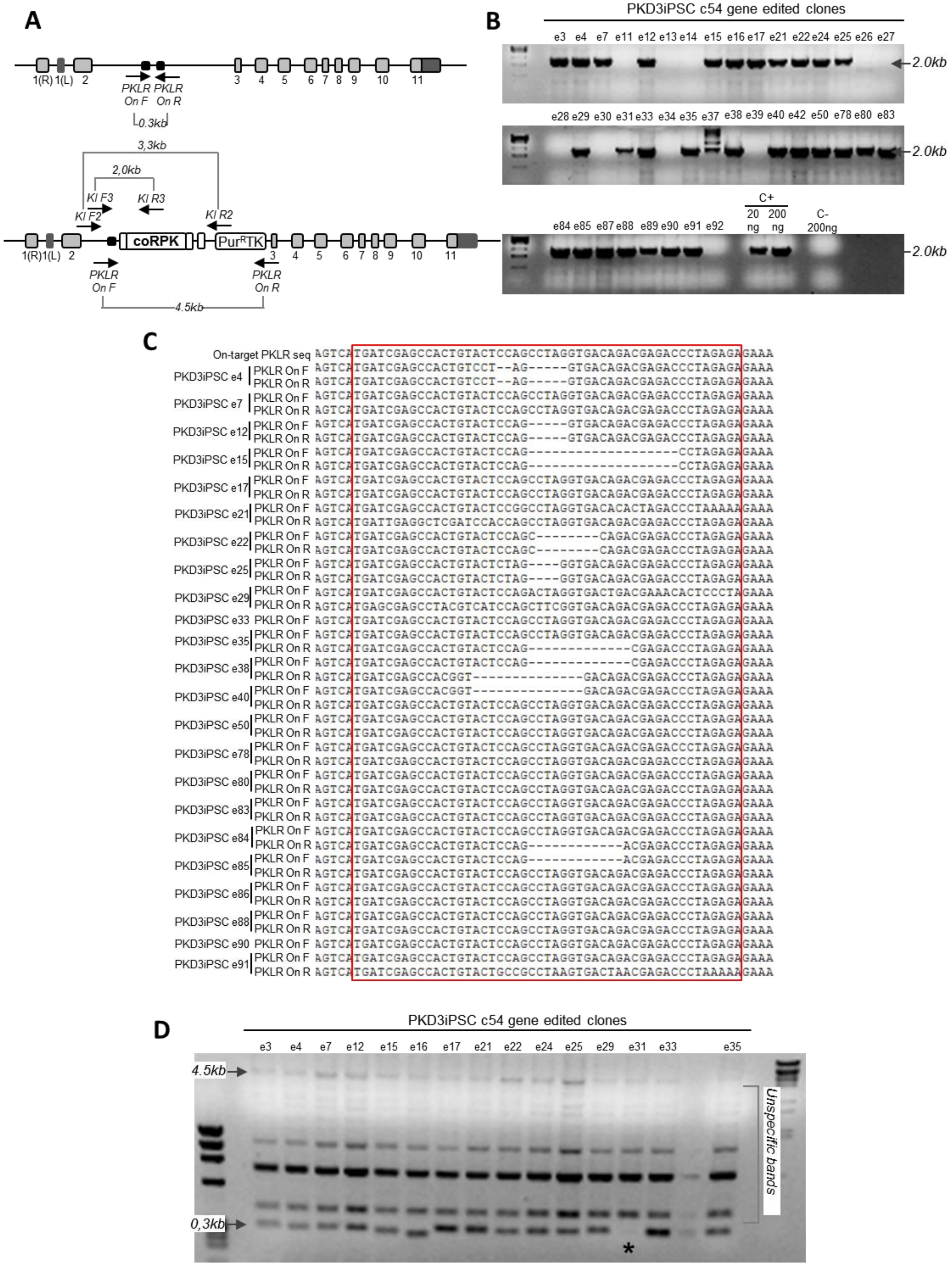
A
1(R) 1(L) 2
PKLR On F
PKLR On R
0.3kb
3 4 5 6 7 8 9 10 11
3,3kb
2,0kb
KI F3
KI R3
KI F2
KI R2
coRPK
Pur^R TK
1(R) 1(L) 2
3 4 5 6 7 8 9 10 11
PKLR On F
PKLR On R
4.5kb
B
PKD3iPSC c54 gene edited clones
e3 e4 e7 e11 e12 e13 e14 e15 e16 e17 e21 e22 e24 e25 e26 e27
2.0kb
e28 e29 e30 e31 e33 e34 e35 e37 e38 e39 e40 e42 e50 e78 e80 e83
2.0kb
e84 e85 e87 e88 e89 e90 e91 e92
C+
20 ng
200 ng
C-
200ng
2.0kb
C
On-target PKLR seq AGTCATGATCGAGCCACTGTACTCCAGCCTAGGTGACAGACGAGACCCTAGAGAGAAA
PKD3iPSC e4 PKLR On F AGTCATGATCGAGCCACTGTCCT--AG-----GTGACAGACGAGACCCTAGAGAGAAA
PKLR On R AGTCATGATCGAGCCACTGTCCT--AG-----GTGACAGACGAGACCCTAGAGAGAAA
PKD3iPSC e7 PKLR On F AGTCATGATCGAGCCACTGTACTCCAGCCTAGGTGACAGACGAGACCCTAGAGAGAAA
PKLR On R AGTCATGATCGAGCCACTGTACTCCAGCCTAGGTGACAGACGAGACCCTAGAGAGAAA
PKD3iPSC e12 PKLR On F AGTCATGATCGAGCCACTGTACTCCAG-----GTGACAGACGAGACCCTAGAGAGAAA
PKLR On R AGTCATGATCGAGCCACTGTACTCCAG-----GTGACAGACGAGACCCTAGAGAGAAA
PKD3iPSC e15 PKLR On F AGTCATGATCGAGCCACTGTACTCCAG-------------------CCTAGAGAGAAA
PKLR On R AGTCATGATCGAGCCACTGTACTCCAG-------------------CCTAGAGAGAAA
PKD3iPSC e17 PKLR On F AGTCATGATCGAGCCACTGTACTCCAGCCTAGGTGACAGACGAGACCCTAGAGAGAAA
PKLR On R AGTCATGATCGAGCCACTGTACTCCAGCCTAGGTGACAGACGAGACCCTAGAGAGAAA
PKD3iPSC e21 PKLR On F AGTCATGATCGAGCCACTGTACTCCGGCCTAGGTGACACACTAGACCCTAAAAAGAAA
PKLR On R AGTCATGATTGAGGCTCGATCCACCAGCCTAGGTGACAGACGAGACCCTAGAGAGAAA
PKD3iPSC e22 PKLR On F AGTCATGATCGAGCCACTGTACTCCAGC--------CAGACGAGACCCTAGAGAGAAA
PKLR On R AGTCATGATCGAGCCACTGTACTCCAGC--------CAGACGAGACCCTAGAGAGAAA
PKD3iPSC e25 PKLR On F AGTCATGATCGAGCCACTGTACTCTAG----GGTGACAGACGAGACCCTAGAGAGAAA
PKLR On R AGTCATGATCGAGCCACTGTACTCTAG----GGTGACAGACGAGACCCTAGAGAGAAA
PKD3iPSC e29 PKLR On F AGTCATGATCGAGCCACTGTACTCCAGACTAGGTGACTGACGAAACACTCCCTAGAAA
PKLR On R AGTCATGAGCGAGCCTACGTCATCCAGCTTCGGTGACAGACGAGACCCTAGAGAGAAA
PKD3iPSC e33 PKLR On F AGTCATGATCGAGCCACTGTACTCCAGCCTAGGTGACAGACGAGACCCTAGAGAGAAA
PKD3iPSC e35 PKLR On F AGTCATGATCGAGCCACTGTACTCCAGCCTAGGTGACAGACGAGACCCTAGAGAGAAA
PKLR On R AGTCATGATCGAGCCACTGTACTCCAG-------------CGAGACCCTAGAGAGAAA
PKD3iPSC e38 PKLR On F AGTCATGATCGAGCCACTGTACTCCAG-------------CGAGACCCTAGAGAGAAA
PKLR On R AGTCATGATCGAGCCACGGT--------------GACAGACGAGACCCTAGAGAGAAA
PKD3iPSC e40 PKLR On F AGTCATGATCGAGCCACGGT--------------GACAGACGAGACCCTAGAGAGAAA
PKLR On R AGTCATGATCGAGCCACTGTACTCCAGCCTAGGTGACAGACGAGACCCTAGAGAGAAA
PKD3iPSC e50 PKLR On F AGTCATGATCGAGCCACTGTACTCCAGCCTAGGTGACAGACGAGACCCTAGAGAGAAA
PKLR On R AGTCATGATCGAGCCACTGTACTCCAGCCTAGGTGACAGACGAGACCCTAGAGAGAAA
PKD3iPSC e78 PKLR On F AGTCATGATCGAGCCACTGTACTCCAGCCTAGGTGACAGACGAGACCCTAGAGAGAAA
PKLR On R AGTCATGATCGAGCCACTGTACTCCAGCCTAGGTGACAGACGAGACCCTAGAGAGAAA
PKD3iPSC e80 PKLR On F AGTCATGATCGAGCCACTGTACTCCAGCCTAGGTGACAGACGAGACCCTAGAGAGAAA
PKLR On R AGTCATGATCGAGCCACTGTACTCCAGCCTAGGTGACAGACGAGACCCTAGAGAGAAA
PKD3iPSC e83 PKLR On F AGTCATGATCGAGCCACTGTACTCCAGCCTAGGTGACAGACGAGACCCTAGAGAGAAA
PKLR On R AGTCATGATCGAGCCACTGTACTCCAGCCTAGGTGACAGACGAGACCCTAGAGAGAAA
PKD3iPSC e84 PKLR On F AGTCATGATCGAGCCACTGTACTCCAGCCTAGGTGACAGACGAGACCCTAGAGAGAAA
PKLR On R AGTCATGATCGAGCCACTGTACTCCAG------------ACGAGACCCTAGAGAGAAA
PKD3iPSC e85 PKLR On F AGTCATGATCGAGCCACTGTACTCCAG------------ACGAGACCCTAGAGAGAAA
PKLR On R AGTCATGATCGAGCCACTGTACTCCAGCCTAGGTGACAGACGAGACCCTAGAGAGAAA
PKD3iPSC e86 PKLR On F AGTCATGATCGAGCCACTGTACTCCAGCCTAGGTGACAGACGAGACCCTAGAGAGAAA
PKLR On R AGTCATGATCGAGCCACTGTACTCCAGCCTAGGTGACAGACGAGACCCTAGAGAGAAA
PKD3iPSC e88 PKLR On F AGTCATGATCGAGCCACTGTACTCCAGCCTAGGTGACAGACGAGACCCTAGAGAGAAA
PKLR On R AGTCATGATCGAGCCACTGTACTCCAGCCTAGGTGACAGACGAGACCCTAGAGAGAAA
PKD3iPSC e90 PKLR On F AGTCATGATCGAGCCACTGTACTCCAGCCTAGGTGACAGACGAGACCCTAGAGAGAAA
PKD3iPSC e91 PKLR On F AGTCATGATCGAGCCACTGTACTCCAGCCTAGGTGACAGACGAGACCCTAGAGAGAAA
PKLR On R AGTCATGATCGAGCCACTGTACTGCCGCCTAAGTGACTAACGAGACCCTAAAAAGAAA
D
PKD3iPSC c54 gene edited clones
e3 e4 e7 e12 e15 e16 e17 e21 e22 e24 e25 e29 e31 e33 e35
4.5kb
0,3kb
Unspecific bands
*

Figure S3, related to Figure 2. Gene editing in *PKLR* locus of PKD3iPSC. (**A**) Diagrams showing the PCR strategy to identify indels and untargeted and targeted alleles together (top) and nested PCR strategy to identify gene edited colonies from PuroR-PKD3iPSC (bottom). Small squares at the beginning and at the end of the coRPK partial cDNA indicate splicing acceptor and flag tag sequences, respectively, present in the cassette; Light gray squares represent endogenous RPK exons; dark gray squares represent first LPK exon and 3'untranslated regions at the beginning and at the end of the PKLR gene, respectively; black squares represent homology arms. (**B**) Nested PCR identifying correct matrix integration in *PKLR* locus. (**C**) Indels in the untargeted allele in edited PKD3iPSC. Sanger sequencing of PCR product to detect indels in the untargeted allele from edited PKD3iPSC. (**D**) Biallelic targeting in edited PKD3iPSC clones. DNA electrophoresis showing PCR products amplified from untargeted and targeted allele. 4.5kb band was detected in all the edited PKD3iPSC clones, this band was not detect in C+, because of this sample is a polyclonal population and the low sensitivity of this PCR. Biallelic targeted PKD3iPSC clones are marked by (*), where 0.3kb band was not detected.

Figure S4

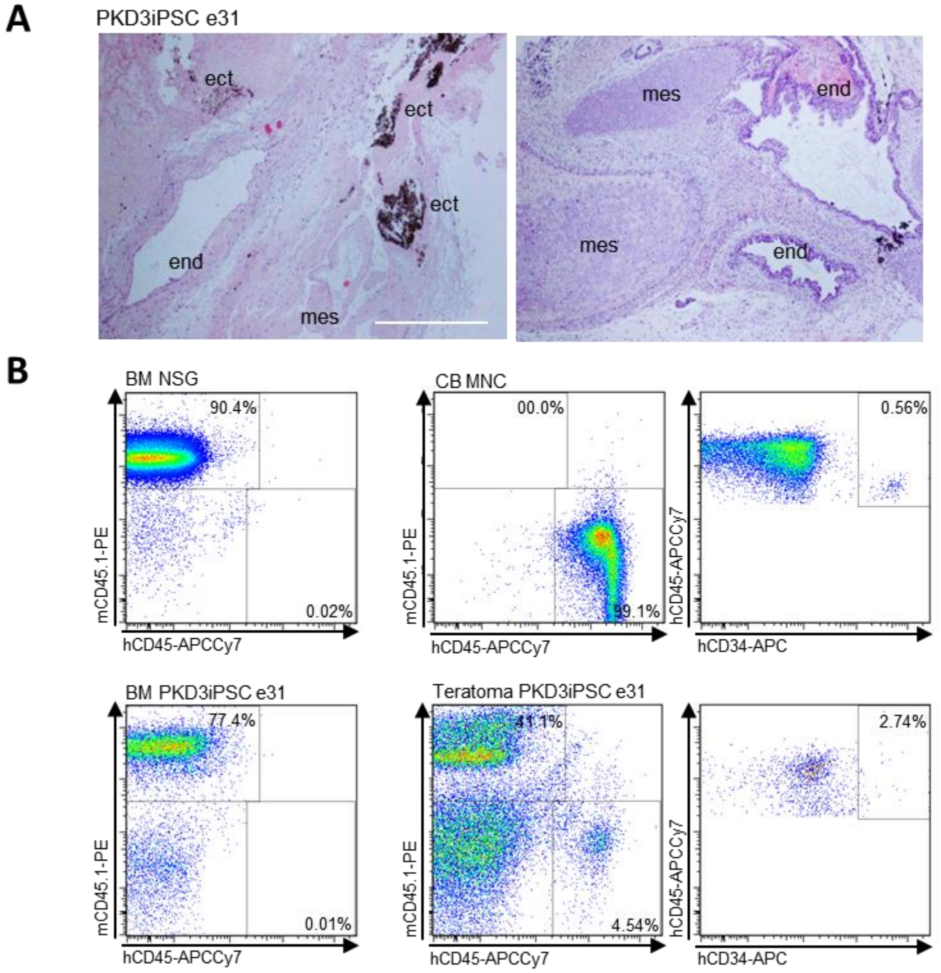

Figure S4, related to Figure 2. *In vivo* pluripotency of edited PKDiPSC. Edited PKDiPSC were injected subcutaneously into NSG mice to evaluate their pluripotency *in vivo*. (**A**) Sections of a teratoma derived from clone PKD3iPSC e31, where tisular structures from the three different embryonic germ layers were observed (ect, ectoderm; end, endoderm; mes, mesoderm). Scale bar 500μm. (**B**) PKD3iPSC e31 were injected into NOD.Cg-*Prkdc*^{scid} *Il2rg*^{tm1Wjl} Tg(CMV-IL3,CSF2,KITLG)1Eav/MloySzJ

(NSG-SGM3) mice and their *in vivo* hematopoietic potential was assessed by flow cytometry. The different dot-plots show: bone marrow from a NSG-SGM3 mouse that has not been injected with human cells (upper left), human cord blood MNC (upper right), bone marrow from NSG-SGM3 mouse that has developed a human teratoma after subcutaneous injection of iPSCs (bottom left) and teratoma analysis for the presence of human hematopoietic cells (bottom right).

Figure S5

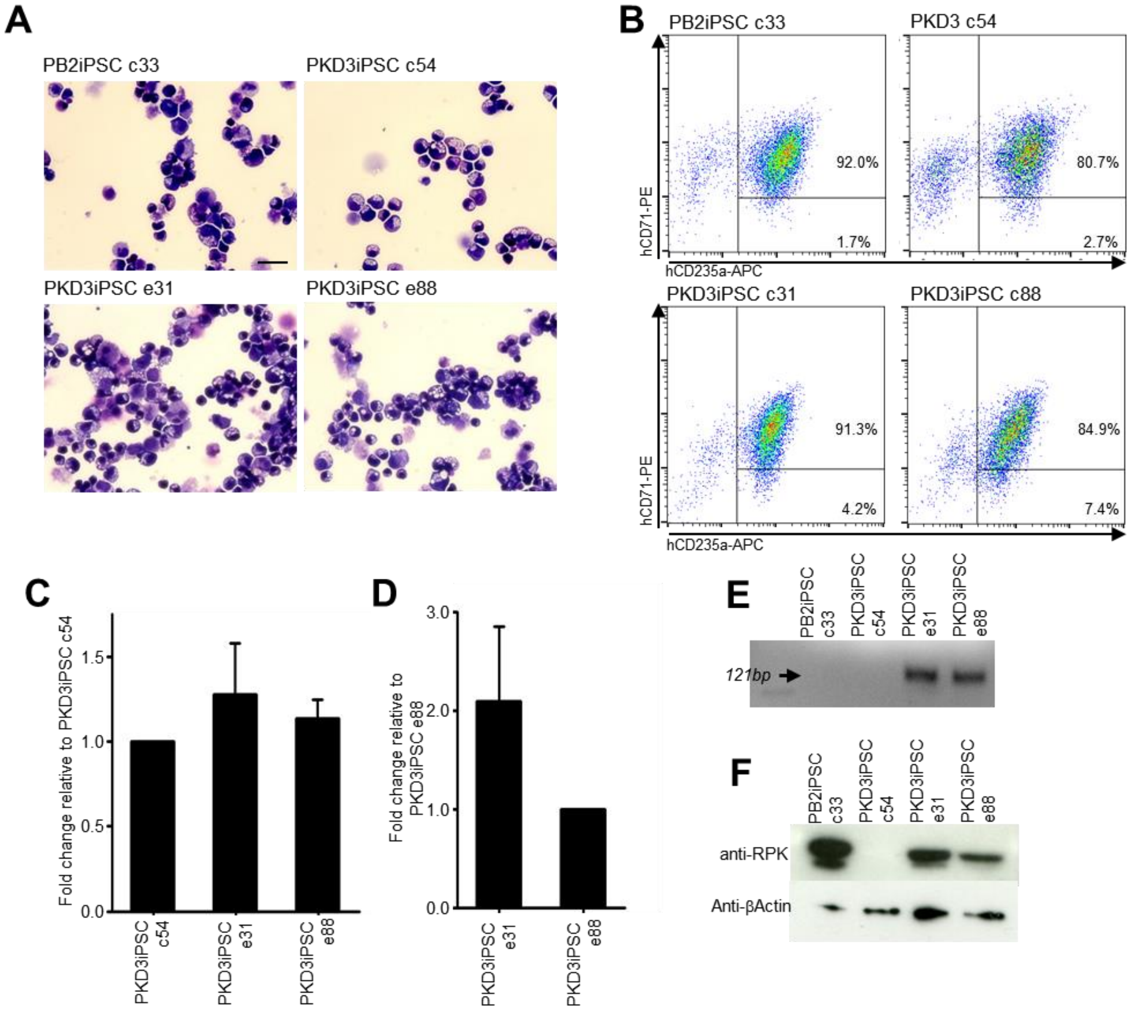

Figure S5, related to Figure 4. Erythroid differentiation of PKD3iPSC. PB2iPSC, PKD3iPSC and monoallelically and biallelically edited PKD3iPSC lines were differentiated to erythroid cells under specific conditions. (**A**) Cell analysis performed by May Grünwald-Giemsa staining. Scale bar 20μm. (**B**) Erythroid differentiation confirmed by flow cytometry analysis. (**C**) RPK expression in erythroid cells derived from the different iPSC evaluated by qRT-PCR. n=3 (**D**) Quantification of chimeric RPK transcript by qRT-PCR. n=3 (**E**) Specific RT-PCR to amplify the chimeric RPK mRNA in edited PKD3iPSC. Arrow indicates the expected band and the corresponding size. (**F**) RPK presence in erythroid cells derived from PB2iPSC, PKD3iPSC and edited PKD3iPSC lines assessed by Western-blot.

Figure S6

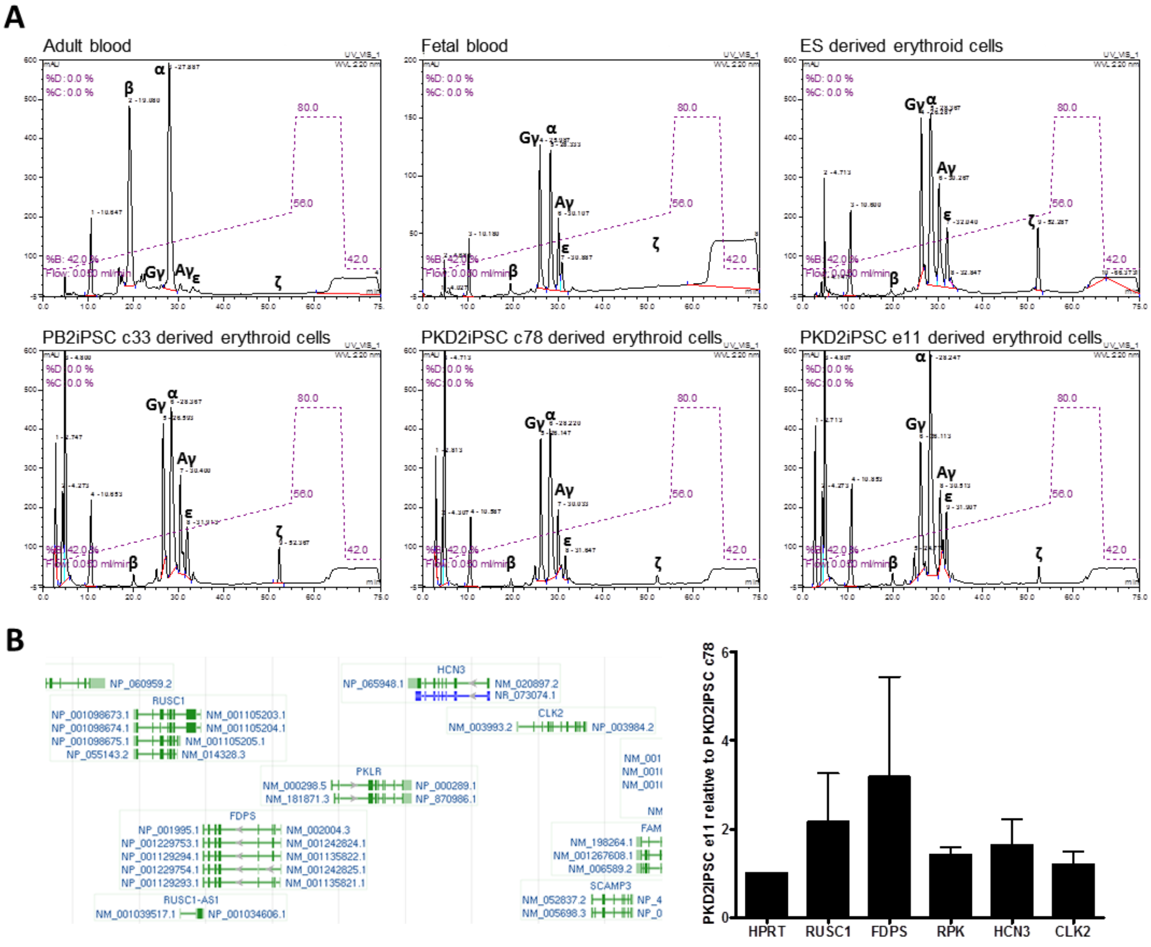

Figure S6, related to Figure 4. Characterization of erythroid cells derived from iPSC. (**A**) Globin pattern analysis of iPSC-derived erythroid cells. Presence of different globins was identified by HPLC in erythroid cells derived from control PB2iPSC, PKD2iPSC and edited PKD2iPSC after 31 days in the differentiation protocol. Adult blood, fetal blood and erythroid cells derived from human ESC were analyzed in parallel as controls of detection of the different globin species. (**B**) Analysis of the expression of PKLR proximal genes in erythroid cells derived from PKD2iPSC c78 and PKD2iPSC e11. PKLR genomic context, indicating their closest genes (left). qRT-PCR analysis to compare the expression of the PKLR proximal genes in erythroid cells derived from PKD2iPSC before and after gene editing (right). No significant differences (p=0.6) were observed. n=3.

Figure S7

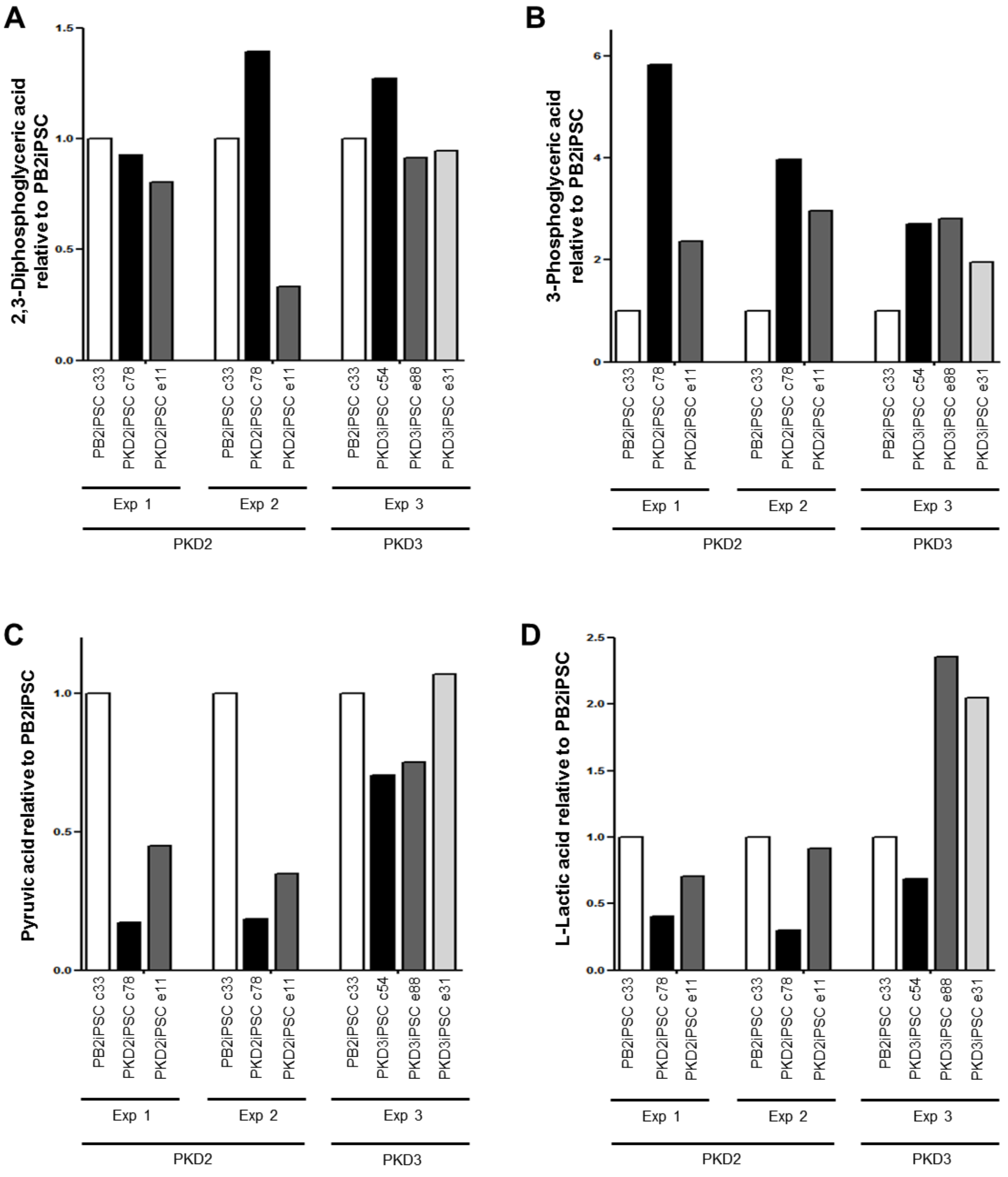

Figure S7, related to Figure 5. Metabolomic analysis of erythroid cells derived from edited PKDiPSC lines. LC-MS analysis of 2,3-diphosphoglyceric acid (**A**), 3-phosphoglyceric acid (**B**), pyruvic acid (**C**) and L-lactic acid (**D**). Data were obtained from erythroid cells obtained from three independent experiments from 6 different iPSC lines coming from two different patients.

Table S1, related to experimental procedures. Primers used.

Name	Sequence
PKD3mut 4F	AGAGACAAGCGATGTCGCCAATG
PKD3mut 4R	TGTTCATGAGGAAAGACAGCAGGCT
Ex4_Fw 2	TTGGGTTTGGTTGCCTCTCA
Ex4_Rv2	TGAGTGGGGAACCTGCAAAG
Ex9_Fw1	GTCCTACAACTTTGACATCC
Ex9_Rv 1	TAGCTCCTCAAACAGCTGC
SeV F	GGATCACTAGGTGATATCGAGC
SeV R	ACCAGACAAGAGTTTAAGAGATATGTATC
KI F1	AGGGTATGCTGAGAGACGAAG
KI R1	CCGGGCGATATTCATGCCG
KI F2	ACTGGGTGATTCTGGGTCTG
KI R2	GGGGAACTTCCTGACTAGGG
KI F3	GCTGCTGGGGACTAGACATC
KI R3	CGCCAAATCTCAGGTCTCTC
PKLR On F	GGTAAATGGCAAAACCCATC
PKLR On R	GCCTGCTTGCTTTCTTTCC
PKLR Off 1 F	CGGGCAGTTGTCGTAATCTT
PKLR Off 1 R	TTTAGCCCAGGAGTTCCAGA
PKLR Off 2 F	CCTGGGCAACAAAATGAGATCTTG
PKLR Off 2 R	CCAACATGGCAAAACCTCATCTCT
PKLR Off 3 F	CTTGAACCCAGGAAGTGGAG
PKLR Off 3 R	CAGTGGCTCACACACGAAGT
PKLR Off 4 F	TCCTGGTAGGATTTTCCACTTCTCA
PKLR Off 4 R	GATGGGGGCAATGGGAAGATT
PKLR Off 5 F	GCACACCCACTGTACTCACCACA
PKLR Off 5 R	GGTGAAACCTCGTCTGCACTGAA
PHF2 F	TGCCATCCTACAGGCTCAAC
PHF2 R	TTGTTTGGAGACGATGCGGA
ZNF747 F	GCTAAGCAGGTAGCTGGAGG
ZNF747 R	CGGGATCCGAGTGGAGAAAG
SNX3 F	ACGTTTTTGCCCCTTCTTGC
SNX3 R	AGTGCATAGCTTTATGGACAGT
TNRC18 F	TTGAGCAGCTCCTTGGCAG
TNRC18 R	CGCTTTTCGGCAAGAAGGAC
TUBGCP6 F	GGGACCCTGTGTGCCAATTA
TUBGCP6 R	TCTGGCTTCTTGTGGATGGG
APOA5 F	TTGCTCAGAACCTTGCCACT
APOA5 R	CTTCCACCCATACGCCGAG
RUSC2 F	TGATGCCAACTGCAACTCCT
RUSC2 R	CAACATACGTTCCAGCGAGC
wtRPK F	GGCCTTGATCATCTCTTTCA
coRPK R	GGAACACCTCTGCCTACTGG
RPK F	ATATCATCCCTGCAGCTTCG
RPK R	CAGCTCCTGGGTCAGTTGG
PKM2 F	CAGCCAAAGGGGACTATCCT
PKM2 R	CCTCAGCCTCACGAGCTATC
HPRT1 F	TGACCTTGATTTATTTTGCATACC
HPRT1 R	CGAGCAAGACGTTCAGTCCT
RUSC1 F	AGGATGGTGCAAACCCATAG
RUSC1 R	CCACTGTGGTGATGACACG
FDPS F	CCCAGGGCAATGTGGATCTT
FDPS R	GCCATCAATTCCTGCCATGT
HCN3 F	CCTCACCGATGGATCCTACTT
HCN3 R	CAGTAGGTGTCAGCCCGAAC
CLK2 F	CATTTAGCCGCTCATCTTCG
CLK2 R	AGTCCCCGACGTGGTAGAT

Table S2, related to Figure 2 and Figure S3. PKLR TALEN off-targets defined by *in silico* studies.

	Refseq	Chromosome	Talen dimer	Left Talen binding site	Left Mismatches	Right Talen binding site	Right Mismatches	NCBI ref seq	start	end
**PKLR On target**	PKLR	1	R-15-L	tctctagggtctcgtc	0	tacagtggctcgatca	0	NT_004487.19	6757643	6757691
**PKLR Off target 1**	no gene	10	L-29-L	tgatTgTgccactgCa	3	tGcagtggcAcAatca	3	NT_030059.13	54947560	54947622
**PKLR Off target 2**	WDR72	15	L-15-L	tgatcgTgccactgCa	2	tGcagtggcAcgatcT	3	NT_010194.17	24745773	24745821
**PKLR Off target 3**	no gene	19	L-28-L	AgatcgTgccactgCa	3	tGcagtggcAcgatcT	3	NT_011295.11	10933216	10933277
**PKLR Off target 4**	no gene	4	L-28-R	tgatGgagcAaTtgta	3	gaGgGgaccctagGga	3	NT_016354.19	91614547	91614608
**PKLR Off target 5**	no gene	6	L-12-L	GgatTgTgccactgta	3	tacagtggcAcgatcT	2	NT_007592.15	26393830	26393875

Capital letters in the off-taget sequences represent mismatches with the on-target ones.

Table S3. Related to Table 2. CNV identified in PKD2 PB-MNC, PKD2iPSC c78 and PKD2iPSC e11.

See Excel file title Table S3.

Table S4. Related to Table 2 and Figure S3. CNVs identified in PKD3iPSC c54, PKD3iPSC e31 and PKD3iPSC e88.

Number	Chromosome	Cytoband	Size (bp)	Type	Present in PKD3iPS c54	Present in PKD3iPS e31	Present in PKD3iPS e88
**1**	1	q31.3	55	AMP	Yes	No	Yes
**2**	2	p11.2	116	AMP	Yes	Yes	Yes
**3**	2	q37.3	130	DEL	Yes	Yes	Yes
**4**	4	q13.2	91	DEL	Yes	Yes	Yes
**5**	6	p25.3	103	DEL	No	No	Yes
**6**	8	p23.1	910	DEL	Yes	Yes	Yes
**7**	8	p11.22	123	DEL	Yes	Yes	Yes
**8**	10	q11.22	1175	DEL	Yes	Yes	Yes
**9**	11	q11	65	DEL	No	No	Yes
**10**	12	p13.31	76	DEL	Yes	Yes	Yes
**11**	14	q32.33	809	AMP	Yes	Yes	Yes
**12**	14	q32.33	398	AMP	Yes	Yes	Yes
**13**	15	q11.1-q11.2	2368	DEL	Yes	Yes	Yes

Table S5. Related to Table 2. Variants identifying by exome sequencing in PKD2 PB-MNC, PKD2iPSC c78 and PKD2iPSC e11. See Excel file title Table S5.

Supplemental Experimental Procedures

iPSC culture and characterization

hiPSC lines were maintained over a layer of irradiated fibroblast, either HFF-1 or mouse embryonic fibroblast (MEF, ATCC) or puromycin resistant MEF (Puro-MEF, STEMCELL Technologies) as required, with hES media, with daily changes. iPSC were passed by either scraping or collagenase IV (Life technologies) treatment. In order to characterize iPSC properties, gene expression of key genes involved in pluripotency and the self-renewal hES was analyzed by hES RT2 Profiler PCR Array as described by manufacturer (SaBiosciences, Qiagen). For immunofluorescence analysis, iPSC colonies were fixed with 4% PFA for 10 minutes and then blocked and permeabilized for 1 hour with PBS/1% BSA/10% FBS/0.3M Glycine/0.1% Tween20. Finally, the colonies were stained by either rabbit anti-NANOG/mouse anti-Tra1-60 or rabbit anti-OCT4/mouse anti-SSEA4 (all from Abcam) and then by a combination of secondary anti Rabbit-Alexa488 (Molecular Probes)/anti Mouse-Texas Red (Jackson Immunoresearch Laboratories) and counterstained by DAPI or Topro3 to visualize cell nuclei. Teratomas were generated by subcutaneous injection of iPSC lines into immunocompromised mice either NOD.Cg-PrkdcscidIL2rgtm/Wjl/SzJ (NSG) or NOD.Cg-*Prkdc*^{scid}*Il2rg*^{tm1Wjl}Tg(CMV-IL3,CSF2,KITLG)1Eav/MloySzJ (NSG-SGM3); once the teratomas were formed, they were removed, formaline-fixed and several sections were stained with hematoxilin/eosin. Detection of patient's mutations was performed by amplifying genomic DNA from PKDiPSC lines surrounding the mutations. PCR products were purified and Sanger sequenced. Clonality at the TCRγ locus was studied using a commercial kit (Master Diagnostica, Granada, Spain), which amplifies genomic TCR γ Gene rearrangements using two primers specific for conserved V and J flanking regions among precursors TcRγδ or TcRαβ lymphocytes Polyclonal (normal donor) and monoclonal (Jurkat or MOLT3) control DNAs were

included for reference. Amplimers were separated and analyzed in an ABI Prism Genetic Analyzer 3110 using GeneMapper V 4.0 from ABI. B-cell clonality at IgH locus was studied using a commercial kit (Master Diagnostica), which amplifies genomic VDJ segments of the hypervariable region at the IgH locus by using specific primer that are complement to constant regions of these regions. Polyclonal (normal donor) and monoclonal sample (RAMOS B cell line) control DNAs were included for reference. Amplimers were separated and analyzed in an ABI Prism Genetic Analyzer 3110 using GeneMapper V 4.0 from ABI. The presence of demethylated CpGs in *NANOG* and *SOX2* promoters was performed by pyrosequencing. SeV genomic RNA analysis was analyzed by RT-PCR using a specific primer pairs that bind SeV large protein.

Plasmids

PKLR TALEN nuclease was synthesized and validated by Cellectis (Paris, France) according to a proprietary protocol. Two plasmids, one of which carrying a PKLR TALEN subunit driven by EF1α promoter to target the second intron of *PKLR* gene were generated. The therapeutic matrix was cloned into a pUC57 plasmid. Briefly, two 1kb homology arms and between them the expression cassette (composed of a splicing acceptor, the codon optimized version of the partial cDNA RPK from exon 3 to exon 11, a FLAG tag and the SV40 polyA) and a floxed selection cassette (formed by the mouse PGK promoter, the puromycin resistance-thymidine kinase fusion gene (PuroR-TK) and the bovine growth hormone polyA (BGH polyA) were introduced. The different parts of the homology matrix were cloned together by In-Fusion HD Cloning kit (Clontech laboratories) following vendor’s guidelines.

Gene editing analysis

PuroR-PKDiPSC colonies were expanded and their genomic DNA was purified analyzed by PCR to detect HR (Figure 2A and Supplementary Figure S8A). To verify biallelic targeting, on-target PCR was modified to allow the amplification of the untargeted and the targeted alleles in the same reaction. For Southern-blot genomic DNA was digested with ScaI and SpeI (New England Biolabs) in separate reactions, transferred to a Nytran™ SuPerCharge nylon membrane by turbo Blotter transfer system (Whatman, GE healthcare) and exposed to UV cross-linking. The DNA probe was generated by excising the HR matrix by HindIII, labeled with radioactive P^{32}dCTPs and then added to the pre-hybridized membrane and incubated overnight using QuikHyb hybridization solution (Agilent). Finally, the membrane was washed to eliminate unbound probe and autoradiographed in an Amersham Hyperfilm ECL (GE Healthcare).

Genetic stability analyses

G-Banding and analysis of metaphasic chromosomes were performed by the Cancer Cytogenetic Core of the Texas Children Cancer and Hematology Center (Houston, Texas). Briefly, cells were incubated overnight with colcemide, treated with 0.075M KCl and a mix 3:1 of methanol/Glacial Acetic Acid and finally dropped into slides and Giemsa stained. The 23 sets of chromosomes were counted in 15-20 metaphasic cells in order to see if there was any chromosome aberration. CGH was performed using the SurePrint G3 ISCA CGH+SNP Microarray Kit (Agilent) by Wicell cytogenetic laboratory (Madison, USA). Bioinformatics analysis was performed using Agilent CytoGenomics Edition 2.5.8.1 and infoQuant CGHfusion 6.1.1. Test samples were compared to Agilent's genotyped reference DNA. It was considered as CNV when the aberration was covered

with more than 3 probes. Exome sequencing was done by Axeq Technologies (MD, USA). Briefly, exome library was generated by SureSelect v4 Exome enrichment kit (Agilent), and then the samples were sequenced for at least 30 fold (30X) on average on target depth coverage using Illumina HiSeq 2000 system with 100-bp paired-end sequencing method. Variant calling and gene annotation were performed as previously described (Ju et al., 2011). In addition, variants had to fulfill these criteria: 1) Read Depth: the number of uniquely mapped reads at the position had to be > 8; 2) The average base quality for the position had to be ≥30; 3) The allele ratio at the position had to be ≥20% for heterozygous variants. The resulting variants that were present also in PKD2 PB-MNC were filtered. No variant from edited PKD2iPSC in which the number of reads in PB-MNC was < 8 was considered as we could not discard their presence in the original population. Variants present in the SNP database (SNPdb) were also removed. Resulting variants were validated by PCR amplification and Sanger sequencing.

Characterization of Erythroid differentiation.

Globins present in differentiated cells were analyzed by HPLC. ATP measure was done using the CellTiter-Glo Luminescent Cell Viability Assay (Promega). To extract cellular metabolites, frozen cells were removed from the -80C° freezer and thawed on a water bath kept at 37°C for 4 minutes. 450 µL of 80% methanol were added to each sample to precipitate the proteins. Samples were then spun at 14,000 rpm for 10 minutes in a centrifuge cooled to 4°C. 50 µL of supernatant was transferred to a 96-well plate and the samples were dried under a nitrogen stream. Extracts were then reconstituted in 50 µL of H_2O containing 1 µg/mL 2-ketobutyric acid-$13C_4$-d_2 internal standard. The plate was put into an autosampler kept at 4°C and 10 µL of each sample was injected onto a tandem Waters UPLC/ABSciEx 4000 QTrap Triple-quadrupole Mass Spectrometer.

The chromatography conditions used were as described by (Buescher et al., 2010). To analyze gene expression, mRNA was purified by TRIzol® (Life Technologies) protocol and retro-transcribed by Ambion RETROscript First Strand Synthesis Kit (Ambion) to obtain cDNAs. qRT-PCR was carried out using Fast SYBR Green Master Mix (Life Technologies). Erythroid cells derived from iPSC were lysed and proteins were extracted to analyze RPK and FLAG tag by standard Western-blot. Briefly, nitrocellulose membranes were incubated with mouse antibody against RPK developed by Dr. N.W. Meza (Táchira Medicine School, University of Los Andes, San Cristóbal, Venezuela) (Diez et al., 2005) or mouse antibody against FLAG (CellSignalling), and then incubated with secondary antibody against mouse conjugated to horseradish peroxidase. Detection was carried out using Amersham ECL Select Western-blotting detection Kit (GE Healthcare) and Kodak Medical X-Ray Films.

Primers

See Supplementary Table S1.

Supplemental References

1. Ju YS, Kim JI, Kim S, et al. Extensive genomic and transcriptional diversity identified through massively parallel DNA and RNA sequencing of eighteen Korean individuals. Nat Genet. 2011;43:745-752.
2. Buescher JM, Moco S, Sauer U, Zamboni N. Ultrahigh performance liquid chromatography-tandem mass spectrometry method for fast and robust quantification of anionic and aromatic metabolites. Anal Chem. 2010;82:4403-4412.
3. Diez A, Gilsanz F, Martinez J, Perez-Benavente S, Meza NW, Bautista JM. Life-threatening nonspherocytic hemolytic anemia in a patient with a null mutation in the PKLR gene and no compensatory PKM gene expression. Blood. 2005;106:1851-1856.
